# Specialized box C/D snoRNPs act as antisense guides to target RNA base acetylation

**DOI:** 10.1371/journal.pgen.1006804

**Published:** 2017-05-24

**Authors:** Sunny Sharma, Jun Yang, Rob van Nues, Peter Watzinger, Peter Kötter, Denis L. J. Lafontaine, Sander Granneman, Karl-Dieter Entian

**Affiliations:** 1 Institute of Molecular Biosciences, Goethe University, Frankfurt am Main, Germany; 2 RNA Molecular Biology and CMMI, F.R.S./FNRS and Université Libre de Bruxelles Rue Profs Jeener & Brachet, Charleroi–Gosselies, Belgium; 3 Institute of Structural and Molecular Biology, Centre for Synthetic and Systems Biology (SynthSys), University of Edinburgh, Edinburgh, United Kingdom; 4 Institute of Cell Biology, University of Edinburgh, Edinburgh, United Kingdom; University of Basel, SWITZERLAND

## Abstract

Box C/D snoRNAs are known to guide site-specific ribose methylation of ribosomal RNA. Here, we demonstrate a novel and unexpected role for box C/D snoRNAs in guiding 18S rRNA acetylation in yeast. Our results demonstrate, for the first time, that the acetylation of two cytosine residues in 18S rRNA catalyzed by Kre33 is guided by two orphan box C/D snoRNAs–snR4 and snR45 –not known to be involved in methylation in yeast. We identified Kre33 binding sites on these snoRNAs as well as on the 18S rRNA, and demonstrate that both snR4 and snR45 establish extended bipartite complementarity around the cytosines targeted for acetylation, similar to pseudouridylation pocket formation by the H/ACA snoRNPs. We show that base pairing between these snoRNAs and 18S rRNA requires the putative helicase activity of Kre33, which is also needed to aid early pre-rRNA processing. Compared to yeast, the number of orphan box C/D snoRNAs in higher eukaryotes is much larger and we hypothesize that several of these may be involved in base-modifications.

## Author summary

Base-pairing of box C/D small nucleolar RNAs (snoRNAs) with the target RNA guides site-specific ribose methylation by fibrillarin (Nop1), a highly conserved methyltransferase. Some box C/D snoRNAs, like yeast U3, U14 and U8 in higher eukaryotes are not involved in methylation but are essential for pre-rRNA processing. Biochemical and computational analyses have led to the identification and characterization of most box C/D snoRNA targets. Several snoRNAs classified as ‘orphan’ snoRNAs lack a guide sequence or have complementarity to RNA that is not characterized. Considering the recent number of studies underscoring association of box C/D snoRNAs with genetic disorders including Prader–Willi Syndrome (PWS), cancer and obesity, it is feasible that apart from ribose methylation and RNA processing, these snoRNAs are involved in several other cellular processes. Here, we report a novel and unexpected function for two yeast orphan box C/D snoRNAs, snR4 and snR45, in site-specific base acetylation. We demonstrate that these box C/D snoRNAs guide cytosine acetylation by Kre33, a factor essential for assembly and processing of the 90S pre-ribosome. Kre33 is known to acetylate a subset of tRNAs with the help of the adaptor protein Tan1. However, here we show Kre33 dependent acetylation of 18S rRNA is guided by orphan snoRNAs. Our results expand the functional repertoire of box C/D snoRNPs and highlight yet another remarkable cellular modularity, where a single enzyme targets different substrates by means of either protein or RNA adaptors.

## Introduction

Non-coding RNA (ncRNA) represents the most abundant form of gene expression in eukaryotic cells [1]. Small nucleolar (sno) RNAs are a group of well-characterized ncRNA molecules of variable length of 60 to 1000 nts. Based on evolutionarily conserved sequence elements, these snoRNAs can be divided into three major classes: the box C/D, box H/ACA and the MRP (Mitochondrial RNA Processing) snoRNAs. These snoRNAs form a scaffold for the assembly of a distinct core of highly conserved proteins to form well-defined C/D and H/ACA snoRiboNucleoProteins (snoRNPs), and the RNAse MRP. Box C/D snoRNPs catalyze site-directed 2′-*O*-ribose methylation, whereas H/ACA snoRNPs catalyze site-directed pseudouridylations of specific rRNA nucleotides [2]. RNAse MRP and some box C/D snoRNAs, like U3, U14 and U8 in higher eukaryotes are involved in pre-rRNA processing. The RNA component of box C/D and box H/ACA snoRNPs functions as an adaptor to guide the catalytic activity of the modification enzyme associated with the RNP to its target site [1].

Canonical box C/D snoRNAs contain conserved and distinctive sequence elements: the C/C′ (5′–RUGAUGA–3′) and D/D′ (5′–CUGA–3′) motifs, and one or two guide sequences of 10–21 nucleotides positioned upstream of the D/D′ regions that can base-pair to the RNA target [2]. These guide sequences direct ribose methylation to the nucleotide base-paired to the 5th nucleotide up-stream of the D or D′ sequence (box D+5 rule) [3]. Target complementarity of these guide sequences can be extended by means of another conserved region located elsewhere in the snoRNA [4]. The 2′-*O*-methylation reaction is catalyzed by the S-adenosyl methionine (SAM) dependent methyltransferase Nop1 (Fibrillarin in higher eukaryotes) [5].

Computational and biochemical analyses have led to the characterization and identification of most box C/D snoRNA targets [6,7]. However, in eukaryotes (including yeast), several snoRNAs appear to lack a guide sequence or have complementarity to rRNA that is not characterized. These snoRNAs are classified as ‘orphan’ snoRNAs. Recent studies have shown that box C/D snoRNAs not only target rRNA and snRNA but also mRNAs and that they may have functions not related to site-directed methylation of ribose sugars [8–10]. In particular, fragments derived from snoRNAs (sdRNAs–snoRNAs derived) have been identified that exhibit miRNA-like characteristics and regulate alternative mRNA splicing in several species including mammals [9,11]. With an increasing number of studies emphasizing the association of box C/D snoRNAs with diseases such as cancer, Prader–Willi Syndrome (PWS) and obesity, it is becoming more likely that these snoRNAs are involved in other cellular processes than we are currently aware of [12–15].

*Saccharomyces cerevisiae* contains 46 box C/D snoRNAs. Apart from three snoRNAs, snR4, snR45 and snR190, the target and/or function of these box C/D snoRNAs have been characterized [16]. Although snR190 contains a guide sequence that could potentially methylate G2395 in the 25S rRNA, no methylation has been reported at this residue [16,17]. As far as snR4 and snR45 are concerned no complementary sequence for any of the rRNAs has been reported. Nevertheless, both snR4 and snR45 have been demonstrated to bind to canonical box C/D snoRNA proteins including Nop1 [4].

Recently, we and others identified and characterized two highly conserved acetylated cytosines in helix 34 and helix 45 of yeast, plant and human 18S rRNA along with their corresponding acetyltransferase—Kre33 (yeast) /NAT10 (human) [18–20]. Kre33 assembles with the pre-rRNA when transcription of the 3′ minor domain (encompassing helices 44–45) has been completed and was proposed to bind as a homodimer [21–23]. In addition, Kre33/NAT10 aided by the adaptor protein Tan1/THUMPD1 acetylates serine and leucine tRNAs [16].

Here, we reveal that the box C/D snoRNAs snR4 and snR45 specifically guide Kre33 to two cytosines that are acetylated in yeast 18S rRNA. CRAC analyses revealed Kre33 binding sites on these snoRNAs as well as on the 18S rRNA (the 5′ domain, helices 34 and 45). We show that both snoRNAs establish extended bipartite complementarity around the targeted cytosines. This base-pairing depends on the putative helicase activity of Kre33 –which we also find to be essential for pre-rRNA processing–and results in looping out of targeted nucleotide.

This is the first demonstration of an unexpected new function of box C/D snoRNPs in directing base modifications. Our data suggest that rRNA acetylation is mechanistically similar to pseudouridylation by H/ACA snoRNPs, where the residue to be modified is isolated and “bulged out” by flanking helices for ready access of the modification enzyme.

## Results

### Kre33 binds both 18S rRNA and the orphan box C/D snoRNAs snR4 and snR45

**Fig 1:**
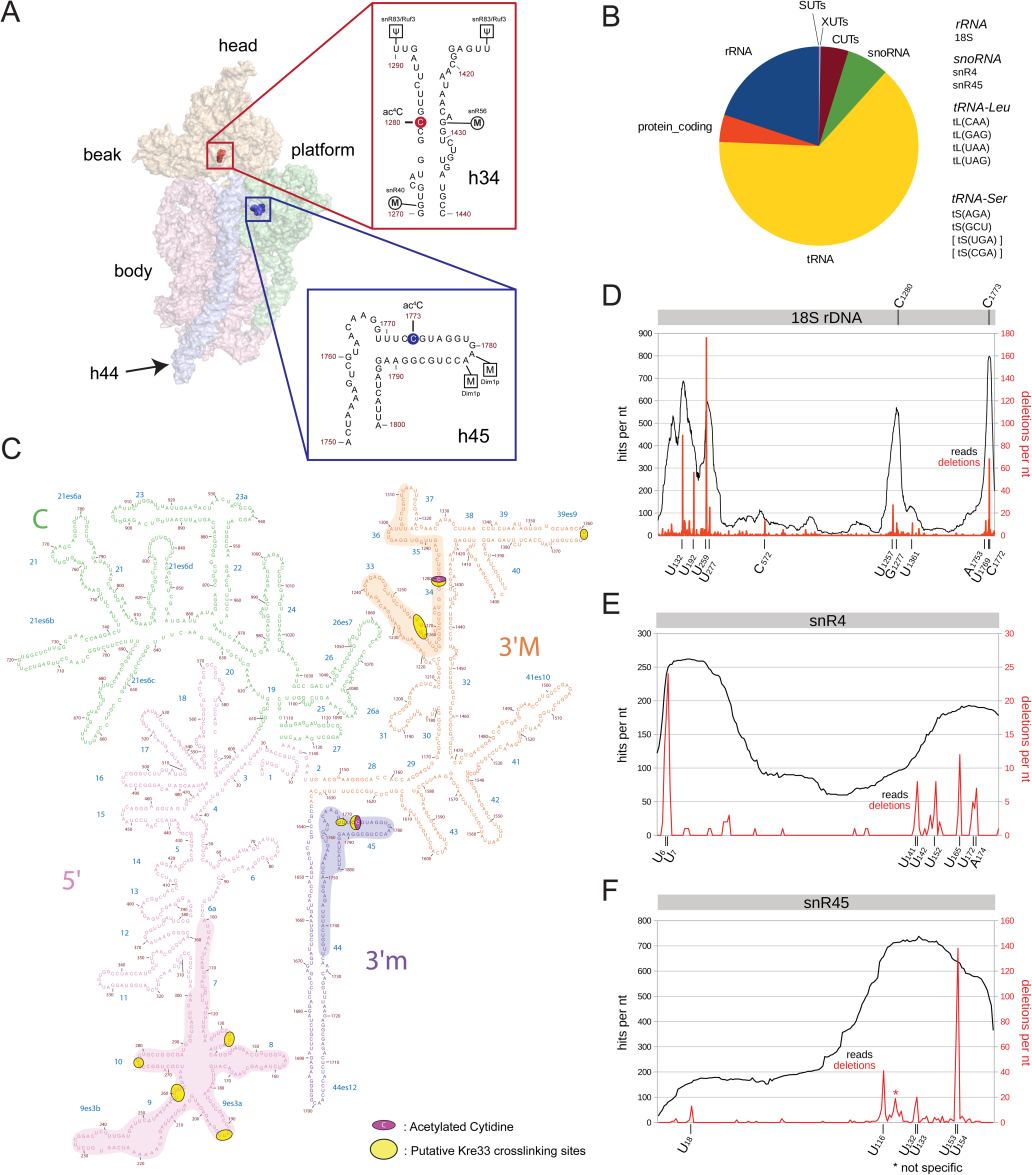
Kre33 forms acetylation complexes with 18S rRNA and snoRNPs snR4 and snR45. A) 3D surface model of the yeast 18S rRNA (3U5B pdb file together with UCSF-Chimera [43] were used to make the surface model). Both acetylated residues in the 18S rRNA, ac^4^C1280 and ac^4^C1773 are highlighted in red and blue spheres, respectively. B) Kre33 binds directly to 18S rRNA, snoRNAs snR4 and snR45 and specifically to leucine and serine tRNAs. Pie-chart representing the relative abundance of various RNA classes in the Kre33 data-set according to FPKMs (Fragments per kilo base transcript per million mapped reads). The RNA species listed on the right specifically cross-linked to Kre33 as found by further data-analysis. Relative abundance of some tRNAs (in brackets) was not above that of those in control CRAC-experiments. C) Kre33 cross linking sites on the 2D structure of 18S rRNA; Kre33 cross-links to the 5′ domain (pink), 3′ major domain (helix 34 (orange)) and 3′ minor domain (helix 44 and helix 45 (blue)). Cross-linked residues in these regions are highlighted in yellow and the acetylated cytosine residues are colored in purple. D-F) Line diagrams showing the total number of hits each time a nucleotide was mapped to the reference sequence (black, left y-axis) and the number of reads with a deletion of that nucleotide (red, right y-axis) plotted against the RNA sequence (x-axis). Apart from 18S rRNA (D), snoRNAs snR4 (E) and snR45 (F) were identified to cross-link to Kre33.

18S rRNA of eukaryotes contains two acetylated cytidine residues, one in helix 34 that is vital for translation fidelity, and another one in helix 45 that constitutes the decoding site of the ribosome (Fig 1A) [18,19]. Acetylation of both cytosines is catalyzed by the highly conserved acetyltransferase Kre33/NAT10 [18–20].

To gain insights into the mechanism of Kre33-directed acetylation, we mapped possible Kre33 rRNA binding sites on the 18S rRNA using CRAC (UV cross-linking and analysis of cDNAs) [24]. Kre33 mainly cross-linked to tRNAs and rRNAs (Fig 1B). Confirming previous work [18], Kre33 cross-linked to leucine and serine tRNAs that are acetylated by Kre33 at position C12 (S1A Fig). Mapping of the cross-linking sites (indicated by high frequency of mutations at specific sites in reads) suggested that in the majority of these tRNAs Kre33 binds proximal to the ac^4^C-12 residue (S1A Fig).

Within the 18S rRNA, Kre33 binds predominantly to the 5′ domain of the 18S rRNA, specifically around helices 7, 8, 9 and 10, making direct contacts with U-residues surrounding positions U132, U192, U259, and U277 of 18S rRNA (Figs 1C, 1D and S1C). Apart from the 5′ domain, Kre33 also cross-linked to helices 34 and 45 proximal to the acetylation sites (Figs 1C and S1B).

**Fig 2:**
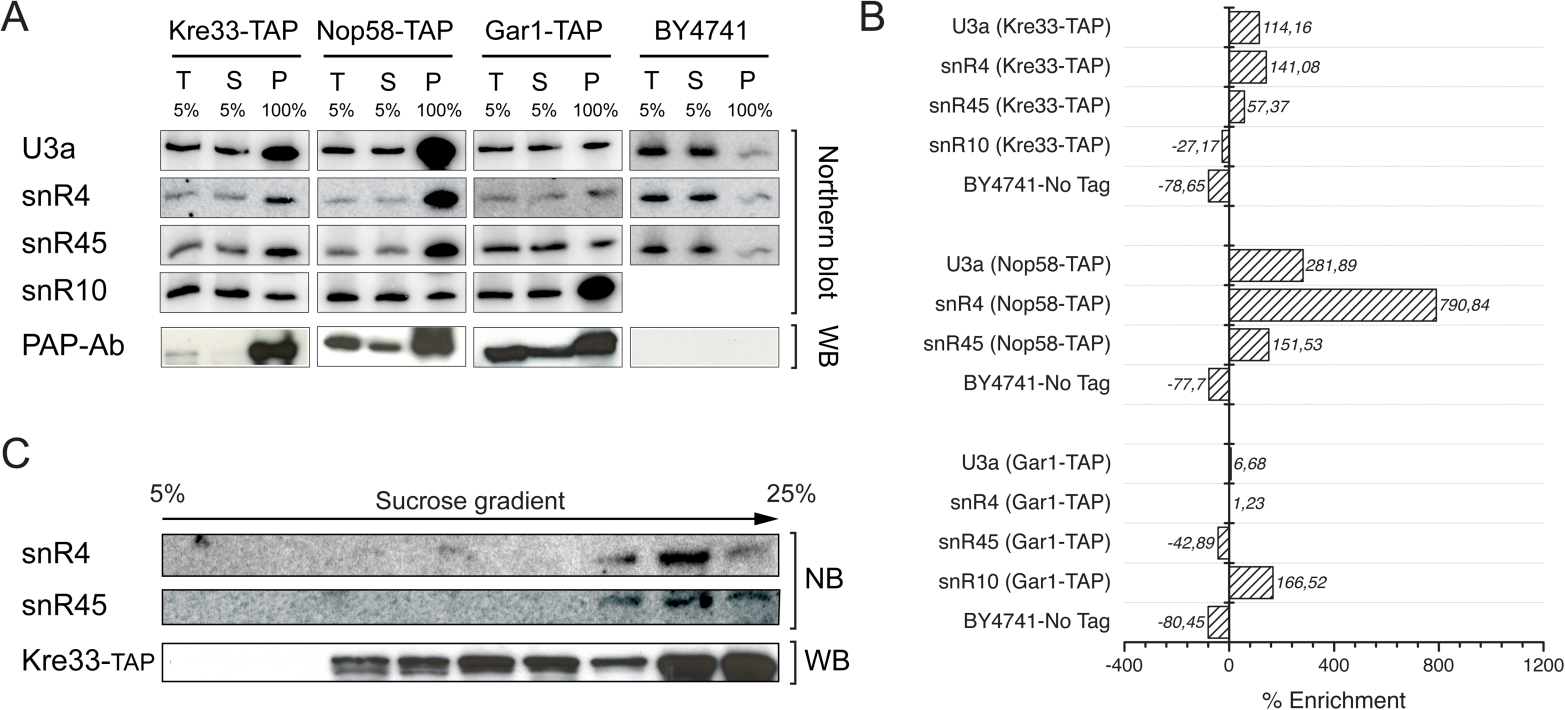
U3, snR4, and snR45 copurify with Kre33. A) RNA isolated from affinity-purified complexes of Kre33, Nop58 and Gar1 TAP-tagged strains were analyzed by northern blotting using probes hybridizing specifically to U3a, snR4, snR45 and snR10. T stands for total cell extract /input, S for supernatant/unbound fraction, and P for pellet /eluate. Mock-purification with the untagged parental strain (BY 4741) was used as a negative control. Nop56 is a core protein of all box C/D snoRNPs, whereas Gar1 is an integral constituent of box H/ACA snoRNPs. These two proteins were used as a positive (Nop58) and negative control (Gar1) for the Kre33-TAP pull-down. B) Quantification of immunoprecipitations with the percent enrichment calculated as the percent change in the signal of respective snoRNA bands in T vs P. Western blot with the PAP antibody exhibiting the specific pull-down of each TAP-tagged protein is shown below the Northern blot panels. C) Co-sedimentation of snR4, snR45, and Kre33 on a 5% to 25% sucrose gradients. Both snR4 and snR45 were detected by Northern blotting and Kre33 by Western blotting using an anti-TAP antibody.

Unexpectedly, we observed significant cross-linking to the orphan box C/D snoRNAs snR4 and snR45 (Figs 1B, 1E, 1F and S1C). The CRAC data showed that Kre33 primarily cross-linked to both the 5′ and 3′ regions of snR4 and to the 3′ end of snR45 (Fig 1E and 1F). Both snR4 and snR45 also co-immunoprecipitated with Kre33-TAP above background levels, confirming the CRAC data (Fig 2A and 2B). Co-precipitation of the box C/D snoRNA U3 (Fig 2A and 2B) is consistent with Kre33 associating with the SSU processome [25]. Fractionation of yeast extracts on density gradients revealed that both snR4 and snR45 co-sedimented with Kre33 in higher molecular weight fractions (Fig 2C), suggesting that Kre33 associates with these snoRNAs in pre-ribosomes. The bulk of Kre33 was detected in lower molecular weight fractions, which presumably represent the population of Kre33 involved in tRNA acetylation (Fig 2C).

### snR4 and snR45 are required for 18S rRNA base acetylation

**Fig 3:**
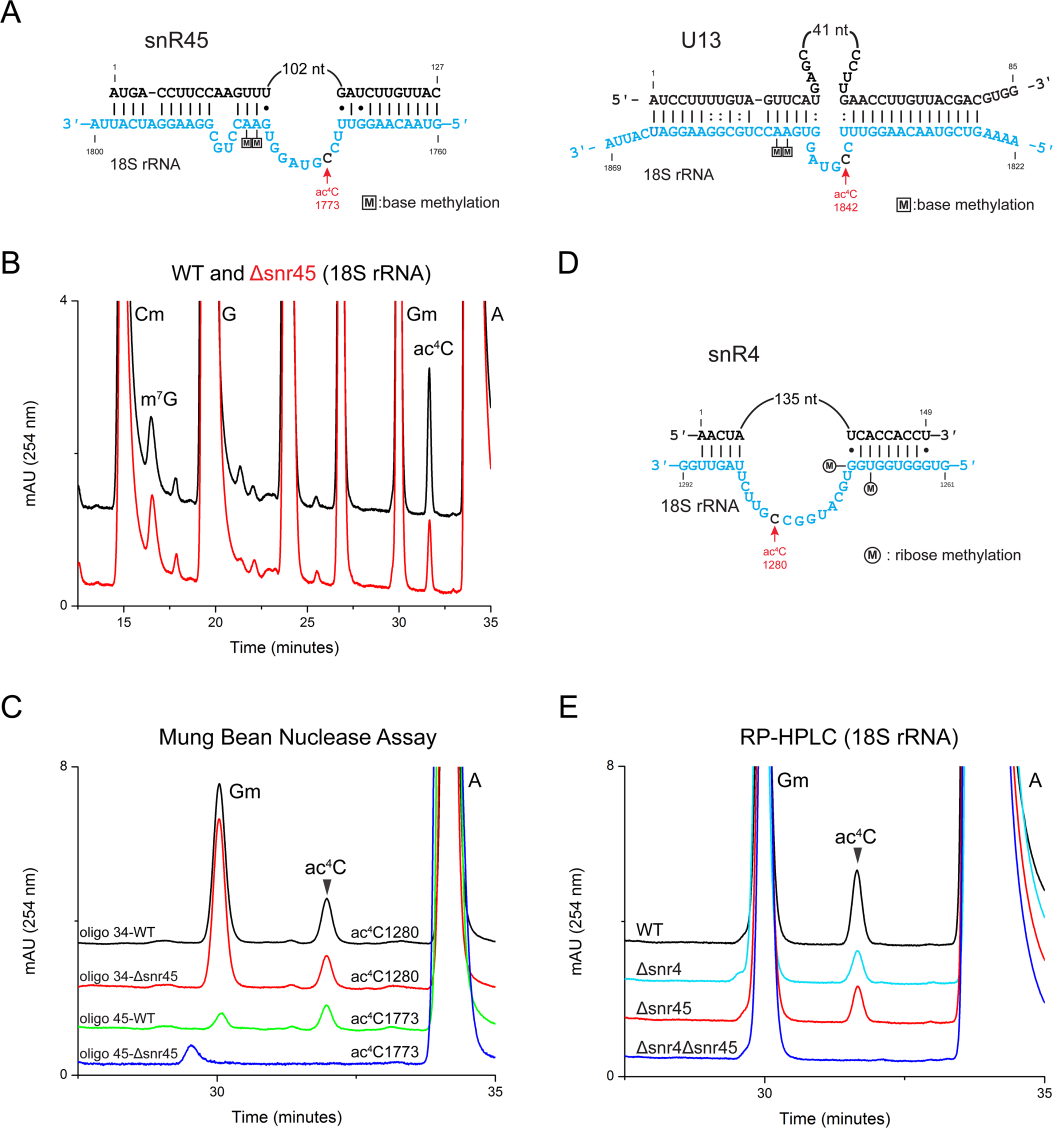
SnR4 and snR45 are distinctly involved in acetylation of 18S rRNA. A) Predicted base-pairing of snR45 and U13 to helix 45 of yeast and human 18S rRNA, respectively. B) Overlaid RP-HPLC chromatograms of nucleosides derived from 18S rRNA of WT (black) and a strain carrying a *SNR45* deletion (Δsnr45, red). C) Overlaid chromatograms of the nucleosides derived from fragments isolated using mung bean nuclease assay, containing ac^4^C1280 (oligo 34) and ac^4^C1773 (oligo 45) isolated from WT (black, green) and Δsnr45 (red, blue). D) Like snR45, snR4 can base-pair to 18S rRNA via extended complementarity to helix 34 proximal to residue ac^4^C1280. E) Overlaid chromatograms of the nucleosides derived from 18S rRNA of WT (black), Δsnr4 (cyan), Δsnr45 (red) and the double mutant Δsnr4Δsnr45 (blue).

Recently, a vertebrate specific U13 box C/D box snoRNA was reported to be involved in 18S rRNA acetylation [18]. However, the precise role of this box C/D snoRNA in 18S rRNA acetylation remained unknown. U13 has been described as a vertebrate or plant specific box C/D snoRNA [26,27]. Conventional bioinformatics software did not allow us to identify any yeast box C/D snoRNA with significant homology to vertebrate U13 [18]. Phylogenetic analysis and systematic comparison of conserved nucleotide sequences of snR4 and snR45, discovered in our CRAC analysis to bind specifically to Kre33, with U13 revealed that snR45 displays a significant sequence similarity to U13 (Figs 3A and S3). Like U13, snR45 showed extended bipartite complementarity to regions around the acetylated cytidine in helix 45 of 18S rRNA indicating that snR45 may act as an antisense guide. These results suggest that snR45 is the likely yeast functional orthologue of the vertebrate and plant U13 snoRNA.

To determine whether yeast snR45, similar to U13, influences 18S rRNA acetylation, we analyzed the nucleotide composition of 18S isolated from a snr45 deletion strain using quantitative RP-HPLC. Loss of snR45 caused a 50% reduction in 18S rRNA acetylation (Fig 3B), suggesting a direct role in the modification of one of the two acetylated cytidine residues that in 18S rRNA of *S*. *cerevisiae* are located at position 1280 in helix 34 and at position 1773 in helix 45 (Fig 1A). To identify the cytosine that remained unmodified in the absence of *SNR45*, we isolated 18S rRNA fragments corresponding to helix 34 and helix 45, respectively, using a mung bean nuclease protection assay. RP-HPLC analyses of these fragments revealed that deletion of *SNR45* leads to complete loss of only ac^4^C1773 in helix 45, whereas ac^4^C1280 in helix 34 remained unaffected (Fig 3C). These results show that yeast snR45 is indeed the functional orthologue of the vertebrate U13 snoRNA and demonstrate that acetylation of the highly conserved cytosine C1773 in helix 45 of the 18S rRNA is dependent on this box C/D snoRNA.

In view of the specific involvement of snR45 in formation of ac^4^C1773, we speculated that the second snoRNA that was enriched in our CRAC data, snR4, could guide the ac^4^C1280 acetylation in helix 34. Like for snR45, conserved guide-like sequences in snR4 were found by phylogenetic comparison. These sequences exhibit extended complementarity to the region around ac^4^C1280 (Figs 3D and S3), which suggested that snR4 assists in acetylation of this residue. Indeed, deletion of *SNR4* led to 50% reduction in the amount of acetylated residues (Fig 3E) and, as found by mung bean protection assay, a complete loss of only ac^4^C1280 without affecting acetylation of ac^4^C1773 (S4A Fig). Upon deletion of both *SNR4* and *SNR45* 18S rRNA cytosine acetylation was completely abrogated (Fig 3E). Because Kre33 also catalyzes acetylation of serine and leucine tRNAs assisted by Tan1, we next determined whether this also depends on the presence of snR4 and snR45. Deletion of both snoRNA genes did not influence tRNA acetylation (S4B Fig), suggesting that snR4 and snR45 are specifically involved in the acetylation of 18S rRNA.

We conclude that the acetyltransferase Kre33 is guided to its two substrate cytosines at positions 1280 and 1773 on the 18S rRNA by the box C/D snoRNAs snR4 and snR45, respectively. Box C/D snoRNAs are known to carry the 2′-*O*-methyltransferase Nop1/Fibrillarin that specifically acts on the sugar moiety of nucleotides. To our knowledge, this is the first demonstration that box C/D snoRNAs can contain more than one modification enzyme, in these cases both a ribose 2′-*O*-methyltransferase (Nop1) and nucleobase acetyltransferase (Kre33).

### snR4 and snR45 utilize H/ACA snoRNA-like bipartite guide sequences for 18S rRNA acetylation

**Fig 4:**
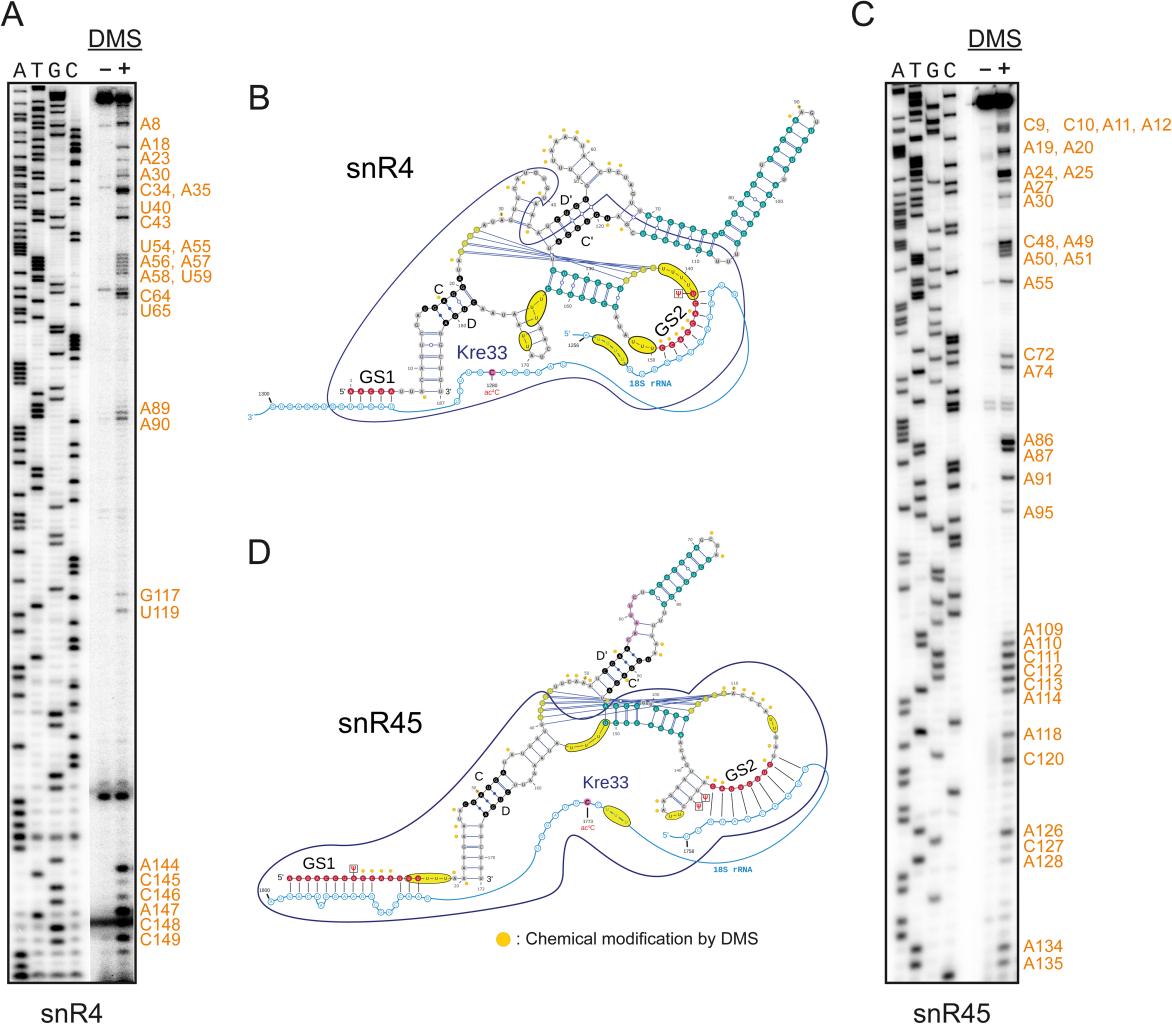
Secondary structures of snR4 and snR45. Secondary structure modeling for snR4 and snR45 was based on phylogenetic analysis (see S2 and S3 Figs) as explained in Materials and Methods, and confirmed by *in vivo* chemical probing. DMS methylated residues were analyzed by primer extension as explained in Materials and Methods. A, C) Representative gels showing structure probing with DMS for A) snR4, and C) snR45. Bands corresponding to modified residues are marked and mapped on to the 2D structure of snR4 (B) and snR45 (D) (orange dots). Shown are the conserved regions with guide sequences GS1 and GS2 (red) and their interactions with 18S rRNA (blue), the C/D and C′/D′ motifs (black), the pseudo-knot (olive) and helices with strong phylogenetic support (teal) as well as modifications on the snoRNAs. Kre33 cross-linking sites identified by CRAC analysis are highlighted (yellow ovals) and the snoRNA-region protected by Kre33 is outlined (dark blue).

To better understand how snR4 and snR45 act in 18S rRNA acetylation, we generated secondary structure models based on *in vivo* DMS RNA structure probing data (Fig 4A and 4C) as well as phylogenetic analysis of snR4 (Figs 4B and S2A) and snR45 sequences (Figs 4D and S3A). DMS predominantly modifies ring nitrogen of exposed A, G and C residues. Secondary structures or poor solvent accessibility protect from DMS methylation [28]. As shown in Fig 4, almost all DMS-modifications were found on nucleotides that have been modelled to be single-stranded or at the termini of proposed helical domains, which we take as supportive evidence for the structures we propose.

Both snoRNAs can adopt structures with a unique albeit comparable architecture. In each snoRNA, the 5′ end consists of a highly conserved sequence with 18S rRNA-complementarity that precedes the C box, while the second guide-like sequence resides in a loop abutting a phylogenetically well-supported helix downstream of the C′ motif. Phylogenetic evidence points to a pseudo-knot formed by this loop and nucleotides downstream of the C box, which would bring both guide regions in close proximity. The putative D′-region is non-canonical in most snoRNAs and not detectable as such in the human or plant counterparts. The bulk of either snoRNA is organized in a variable helical region that bridges the D′ and C′ motifs. With these secondary structures, we can begin to model how the bipartite base-pair interactions between 18S rRNA and the guide-sequences of snR4 and snR45 can expose the cytidine residue that has to interact with acetyltransferase domain of Kre33 (Fig 4B and 4D). Kre33 protects predominantly the snoRNA regions involved in these base-pair interactions and mapping of its cross-linking sites on the 2D structure of snR4 and snR45 revealed that Kre33 makes a direct contact proximal to the guide sequence in the loop (Fig 4B and 4D). Notably, the Kre33 binding sites on 18S rRNA are spatially adjacent to those of snR4 and snR45, consistent with the idea that these orphan snoRNAs guide Kre33 to its target sites.

**Fig 5:**
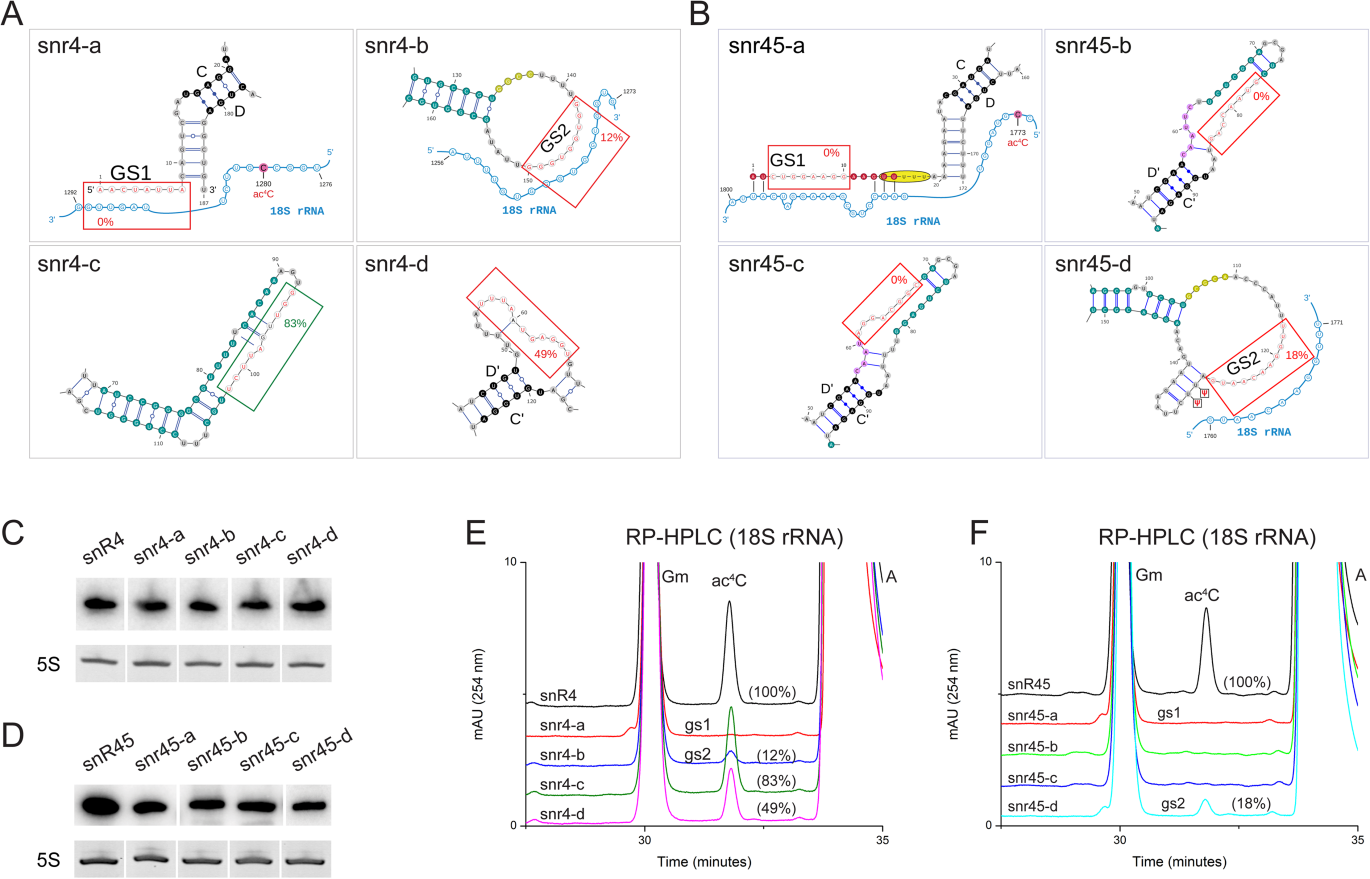
Physical interaction of snR4 and snR45 with 18S rRNA is indispensable for acetylation of C1280 and C1773. Mutations of snR4 (A) and snR45 (B) that were tested are boxed with the resulting acetylation efficiency indicated (%; red for negative; green for neutral; substituted nucleotides are red on white). Mutant snoRNAs were stably expressed as shown by Northern blot developed with a snoRNA-specific probe, C) for snR4 and D) for snR45. E) RP-HPLC chromatograms of 18S rRNA isolated from strain Δsnr4Δsnr45 expressing mutant snr4-a (red), -b (blue), -c (green) or -d (pink). Acetylation efficiencies are in brackets. F) RP-HPLC chromatograms of 18S rRNA isolated from strain Δsnr4Δsnr45 expressing mutant snr45-a (red), -b (green), -c (blue) or -d (cyan). Acetylation efficiencies are in brackets. 5S rRNA was used as loading control.

We tested whether the guide sequences, referred from here onward as GS1 (at the 5′) and GS2 (in the loop), are essential for acetylation. GS1 of snR4 extends from nucleotide number 1 to 5 and according to our model establishes base pairing with nucleotides 1286 to 1290 of 18S rRNA (Fig 4B). GS2 consists of nts 142 to 149 and interacts with 18S nts 1264 to 1271 (Fig 4B). Similarly, GS1 of snR45 covers nts 1 to 15 that base-pair to nts 1781 to 1798 of 18S rRNA (Fig 4D); GS2 includes nts 120 to 127 of snR45 and binds to nts 1760 to 1767 of 18S rRNA. Along with other conserved regions (shown in Figs 5A, 5B, S6A and S6B), we mutated these sequences in both snR4 and snR45 (Figs 5A, 5B, S6C and S6D) and expressed mutant sno-RNAs from plasmids in a strain with a double deletion for *SNR4* and *SNR45*. Mutant snoRNAs that were stably expressed were tested for functionality (acetylation) *in vivo* (Figs 5C, 5D and S6G).

Disruption of the base-pairing between GS1 of snR4 and snR45 with 18S rRNA resulted in complete loss of acetylation at residue C1280 and C1773, respectively (Fig 5E and 5F). Similarly, disrupting GS2 of both snoRNAs resulted in 88% and 82% reduction in acetylation at residue C1280 and C1773, respectively (Fig 5E and 5F). These observations demonstrate that the guide sequences GS1 and GS2 are essential for efficient acetylation.

Apart from guide sequences, different conserved regions of both snR4 and snR45 (highlighted in Figs 5A, 5B and S6A–S6D) were altered. The effect of these mutations on acetylation at position 1280 or 1773 (Fig 5E and 5F) indicates that the helical segments that maintain the architecture of the snoRNAs are important (snR4) or essential (snR45) for their function in guiding acetylation, and this does not simply reflect a requirement for snoRNA stability, but presumably their impact on higher order structure of the snoRNAs (Figs 5C, S6E, 5D and S6F).

We also attempted to introduce compensatory 18S rRNA mutations in helix 34 and 45 to determine if the observed acetylation defects in snR4 and snR45 could be rescued. Every single mutation we generated in helix 34 or 45, however, was lethal.

We conclude that the predicted snR4 and snR45 guide sequences are essential for acetylation of 18S rRNA at C1280 and C1773. Corroborating the involvement of these snoRNA in guiding acetylation, we found, among hybrid RNAs crosslinked to Kre33, a chimera of snR4 and its proposed binding site on 18S rRNA (see below).

### Putative helicase activity of Kre33 facilitates annealing of snR4 and snR45 to the 18S rRNA

**Fig 6:**
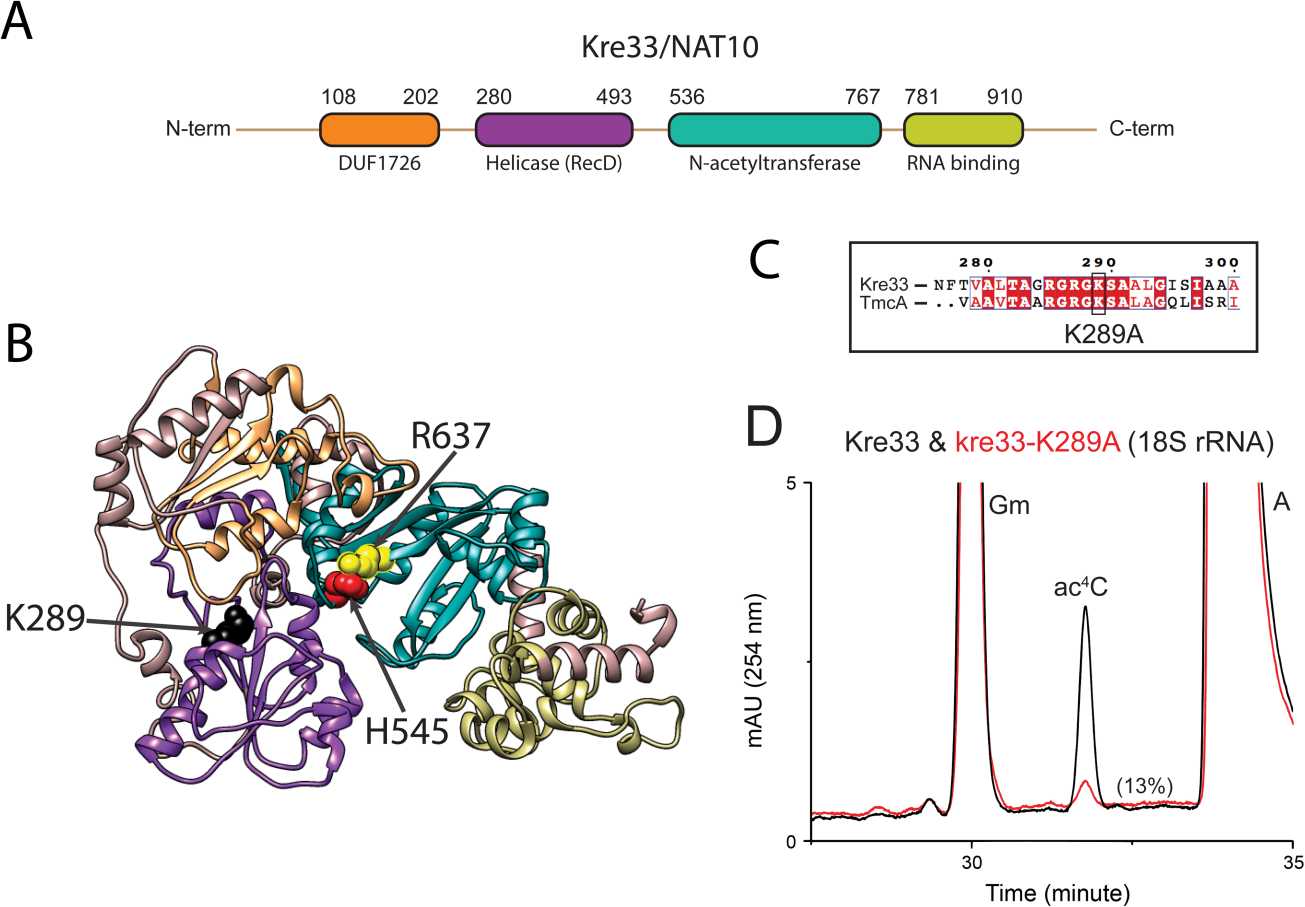
Putative Helicase activity of Kre33 is vital for 18S rRNA acetylation. A) Domain architecture of Kre33. B) *In silico* 3D structure of Kre33, predicted using Phyre [42] and prepared using UCSF Chimera [43]. C) The Walker A motif (P loop) in the helicase domain of Kre33 and TmcA is highly conserved. The substitution of lysine 289 to alanine (K289A) in Kre33 leads to a dramatic loss of 18S rRNA acetylation. D) Overlaid chromatograms of the nucleosides derived from 18S rRNA of WT and the helicase mutant (K289A).

**Fig 7:**
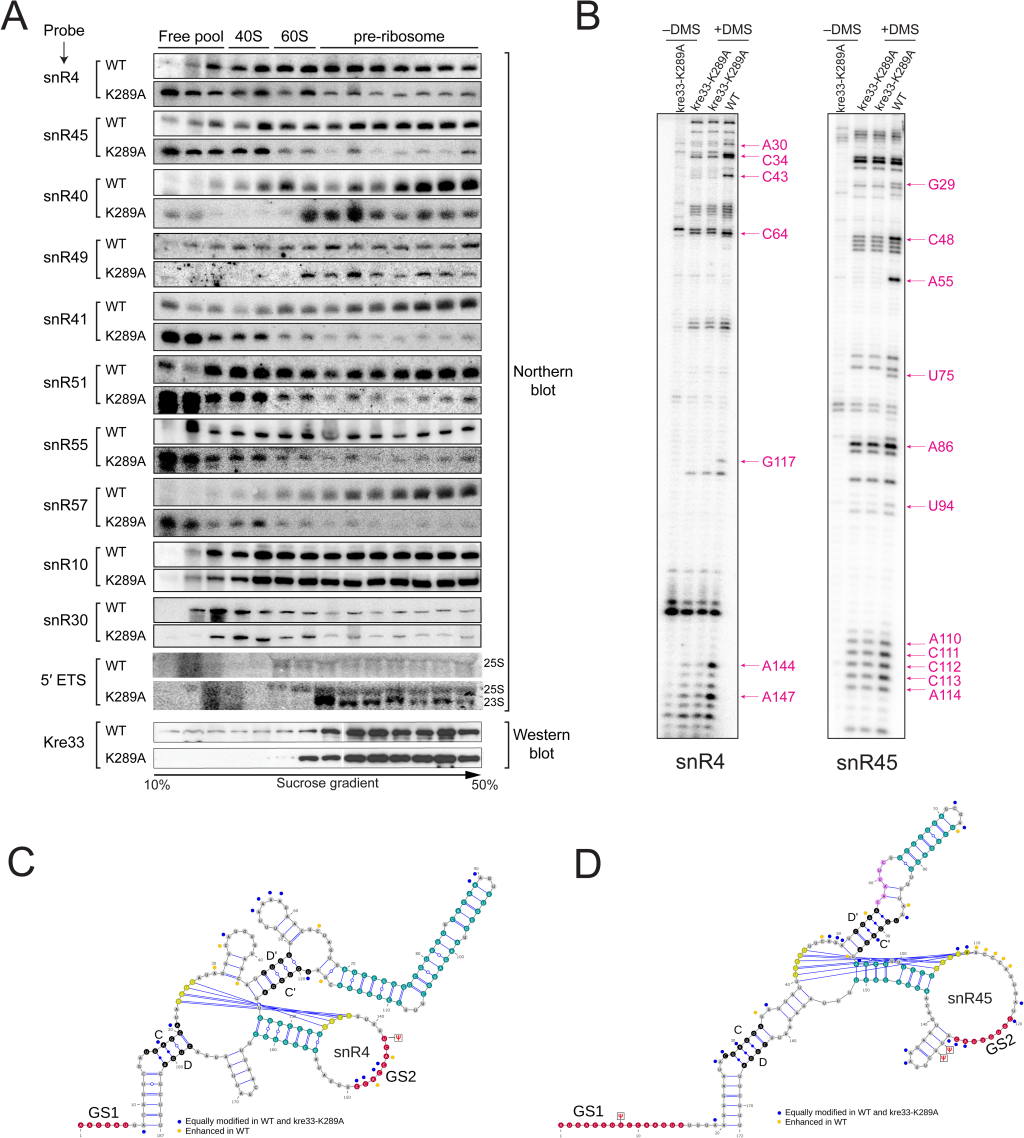
Loss of putative helicase activity affects the binding of snoRNAs in pre-ribosomal complexes. A) Sucrose gradient distribution of snR4, snR45, snR40, snR55, snR49, snR51, snR57, snR41, snR10, snR30 and aberrant 23S rRNA in the isogenic WT and helicase mutant (K289A). All snoRNAs were detected by Northern blot using specific probes and WT-Kre33 and kre33-K289A on a Western blot with anti-His antibody (Qiagen). Aberrant 23S rRNA was detected by Northern Blot using a probe specific to 5′ETS (cf. Fig 8A). Loss of helicase activity of Kre33 affects the structure of snR4 and snR45. B) DMS structure probing of snR4 and snR45 in the WT and the K289A mutant (two independent experiments). Bands corresponding to nucleotides with altered (orange dots) and unaltered (blue dots) DMS reactivity are annotated and mapped on the 2D structures of snR4 (C) and snR45 (D).

In addition to its acetyltransferase domain, Kre33 contains an N-terminal DEAD-box like helicase module (Fig 6A). Changing the conserved lysine residue in the Walker A motif (P loop) of the Kre33 helicase domain to an alanine (K289A) (Fig 6B, 6C and 6D) leads to a ~90% reduction in 18S rRNA acetylation (Fig 6D) [16]. Notably, the corresponding lysine residue in the bacterial homolog of Kre33, TmcA (Fig 6C), has been shown to be indispensable for its ATPase-dependent helicase function [29]. How the helicase domain of Kre33 influences the acetylation reaction remains unclear. Helicases, including DEAD-box helicases, not only catalyze unwinding of RNA duplexes, but can also facilitate strand annealing [24–26]. Because the kre33-K298A mutant did not support normal acetylation, it seems unlikely that Kre33 is required for dissociation of snR4 and snR45. We therefore hypothesized that the helicase domain of Kre33 facilitates the binding of snR4 and snR45 to their respective targets. To test this, we analyzed the distribution of snR4 and snR45 in cell lysates fractionated by sucrose density gradient centrifugation. Cell extracts were prepared from strains expressing the wild-type Kre33 or the kre33-K289A helicase mutant (Fig 7A). Interestingly, while the K298A mutation did not strongly impact the distribution of Kre33 in the gradient, it significantly increased snR4 and snR45 levels in the lower molecular weight fractions (Fig 7A). We also observed a similar profile for snR4 and snR45 upon Kre33 depletion (S7 Fig). In contrast the helicase mutation did not noticeably affect Kre33 co-sedimentation with higher-order complexes (Fig 7A). These data suggest that the predicted Kre33 helicase activity is not important for association with 90S pre-ribosomes, but it is important for efficient recruitment of these snoRNAs to 90S pre-ribosomes. A significant reduction in DMS reactivity of snR4 and snR45 in K289A mutant further suggests a decrease in Kre33 interaction with snR4 and snR45. This interaction with Kre33 is likely vital for both snoRNPs to attain a conformation that is necessary for their interaction with pre-ribosomes.

**Fig 8:**
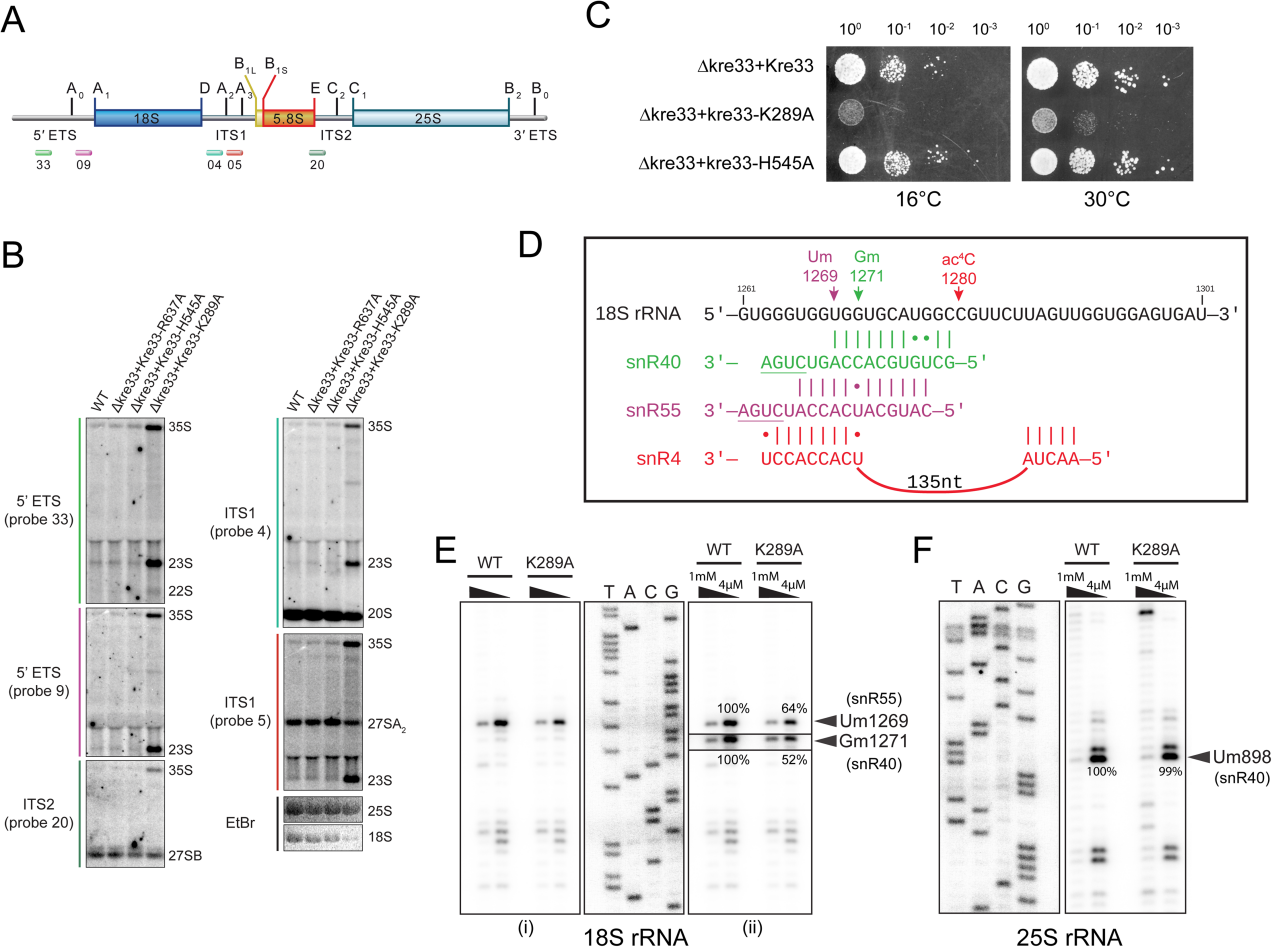
Loss of putative helicase activity of Kre33 affects early pre-rRNA processing and 18S rRNA modification by snR40 or snR55. Early pre-rRNA processing requires the predicted helicase activity of Kre33 and does not depend on acetylation. A) Overview of the 35S primary transcript. 35S pre-rRNA contains 18S, 5.8S and 25S rRNA sequences separated by internal transcribed spacers (ITS1 and ITS2). B) Northern blot analysis of pre-RNA processing in WT and strains expressing mutant Kre33 in which its helicase (K289A) or acetylation (H545A, R637A) activity was abolished. The membrane was hybridized with radioactively labeled probes annealing to 5′ ETS, ITS1, or ITS2 sequences. The increased levels of 35S pre-rRNA and the accumulation of the aberrant 23S species in the case of kre33-K289A are indicative for reduced processing at sites A_0_, A_1_ and A_2_ (which is bypassed by cleavage at A_3_) resulting in very low steady state levels of 18S rRNA (EtBr panel). In line with defective 18S formation, absence of putative Kre33-helicase activity leads to a significant growth defect (C). D) Guide-sequence interaction of snR40 (green), snR55 (magenta) and snR4 (red) with 18S rRNA (black). The target sequences of these snoRNAs in h34 of 18S rRNA overlap with each other. Primer extension analysis of ribose methylation in helix 34 of 18S rRNA (E) and helix 33 of 25S rRNA (F). ^32^P-labeled primer complementary to nucleotides 1315 to 1336 of yeast 18S rRNA (E) and to nucleotides 947 to 967 of 25S rRNA (F) were used for methylation analysis of Um1269 (snR55) and Gm1271 (snR40) (E) in the 18S rRNA and Um898 (snR40) (F) in the 25S rRNA. Since the bands corresponding to Gm1271 were barely visible in comparison to Um1269 (E(i)), the levels for these bands were altered in the boxed section (E(ii)). Bands corresponding to Um1269 and Gm1271 were quantified using ImageJ software (http://imagej.nih.gov/ij/).

Our rRNA processing analysis of the helicase mutant revealed that loss of putative helicase activity impairs early A_0_, A_1_ and A_2_ cleavages, leading to an accumulation of aberrant 23S and 22S rRNA species, a pattern that was previously observed upon hypomorphic expression of Kre33 (Fig 8B) [16]. We also observed a significant growth defect in the helicase mutant (K289A) compared to isogenic wild type and the acetylation deficient mutant (H545A) of Kre33 (Fig 8C).

This prompted us to test the sedimentation profile of other snoRNAs–snR40, snR51, snR55, and snR49, snR57, and snR41 –in kre33-K289A, including those that bind in the vicinity of Kre33 binding sites on the 18S rRNA (snR40, snR55, snR51, and snR49). Sedimentation profiles of snR41, snR57, snR51 and snR55 in the helicase mutant appeared very similar to those of snR4 and snR45 (Fig 7A), suggesting that putative helicase activity of Kre33 is required for the association of both snR51 and snR55 with 90S pre-ribosomes. On the other hand, the sedimentation profiles of snR40, and snR49 revealed that these snoRNAs accumulate in fractions with aberrant 23S pre-rRNA containing pre-ribosomes (Fig 7A). This suggests that the putative helicase activity of Kre33 is necessary for efficient release of these snoRNAs from the pre-ribosome. The sedimentation pattern of snR10 and snR30, snoRNAs that are not associated with Kre33 containing 90S [22] remained unchanged in the helicase mutant (Fig 7A).

Intriguingly, the annealing sites for snR40 and snR55, which modify Gm1271 and Um1269 in helix 34 of 18S rRNA, overlap with the GS2 base-pairing site of snR4 and Kre33 (Fig 8D). We found 2′-*O*-methylation at these sites to be significantly reduced in the kre33-K289A mutant (Fig 8E and 8F). Considering the different sedimentation patterns of snR40 vs snR4 and snR55 in the Kre33 helicase mutant, this would imply that snR40 may remain bound on pre-rRNA, which in turn could block the association of snR4 or snR55. Therefore, it is possible that Kre33 is required for facilitating the release of snR40 and the subsequent association of snR4 and/or snR55. It is equally possible that in view of the strong processing defect in absence of Kre33 that other, essential assembly/processing factors rely on putative Kre33 helicase activity for their function or release. Therefore, the dependence on putative helicase activity of Kre33 for acetylation and other modifications could be indirect, namely as a consequence of upstream 90S conformational changes that are facilitated by Kre33.

We analyzed our CRAC-data-sets with the Hyb-pipeline [30] to find chimeric reads of different RNAs that were cross-linked to the same Kre33 molecule and therefore could have been ligated together during library preparation [31]. In direct support of a role of snR4 during acetylation by Kre33, we retrieved one hybrid between GS2 of snR4 and its predicted target region in helix 34, overlapping the snR40 and snR55 methylation sites (S1 Table, Fig 8D). Providing further evidence for a role of Kre33 in controlling snoRNA occupancy on pre-rRNA the data-set contained two hybrids of snR40, one with its target site in helix 34, the other around Gm562, a recently discovered snR40 target [32]; six hybrids between snR77 and the nearby methylation site at Um578; one hybrid of snR52 and one of snR79 with their annealing sites over Am420 and Cm1007, respectively; and two hybrids of U14 with a region around position 100, while, notably, seven hybrids of snR55 with its target region in helix 34 were found (S1 Table). At all these sites but the one for snR79 we detected Kre33 binding (S1 Table). Apart from many rRNA-rRNA hybrids, we retrieved hybrids of the 25S rRNA region around 900: one with snR4, overlapping the target-site of snR40 and near that of snR60 for which two hybrids were retrieved. Furthermore, two hybrids for snR45 with a 3′ region of 25S were found. Whether these observations point to a direct role of Kre33 in the regulation of snoRNA-25S interactions and the timing of pre-rRNA processing remains to be determined.

## Discussion

Functional analyses of non-coding RNAs including snoRNAs have revealed that the majority of these ncRNAs act as a scaffold for the assembly of a catalytic complex and are responsible for the substrate specificity [1]. Although the large majority of the box C/D snoRNPs catalyze site-specific ribose methylation, a few are known to be involved in other processes, such as rRNA processing and regulation of alternative mRNA splicing [1,33]. Herein, we expand the functional repertoire of the box C/D snoRNAs to site-specific base acetylation. Recently, we identified two highly conserved acetylated cytidines, one in helix 34 and another one in helix 45, in the 18S rRNA of yeast, plant and human, catalyzed by a highly conserved acetyltransferase, Kre33/NAT10. In the present study, we showed specific involvement of two orphan sno-RNAs, snR4 and snR45, in guiding Kre33-dependent acetylation of budding yeast 18S rRNA: snR4 for ac^4^C1280 and snR45 for ac^4^C1773.

Our analysis of the guide sequences for both snR4 and snR45 revealed that in contrast to the canonical box C/D snoRNAs, snR4 and snR45 function akin to H/ACA snoRNA where the guide sequences establish base-pairing with the regions on either side of the target nucleotide and result in looping out of this target nucleotide. Both snR4 and snR45 adopt a comparable fold and establish base-pairing in a similar fashion with the 18S rRNA on either side of the acetylated residue which leads to the fixation of a 9 to 11 nucleotides long bulge that contains the targeted cytosine (Fig 4B and 4D). This looping out facilitates the accessibility of the targeted base to Kre33 and appears to be a salient feature of snoRNAs involved in modifying a nitrogenous base: isomerization in the case of H/ACA snoRNPs and acetylation in the case of snR4 and snR45. Nevertheless, unlike for ribose methylation and pseudouridylation, it is very difficult to establish the sequence rules for snR4 and snR45. This is primarily because so far, we have only encountered two such specialised snoRNPs.

Apart from guide sequences that target snR4 and snR45 for Kre33 mediated acetylation, these snoRNAs contain canonical C/D and C′/D′ boxes with a complete set of core C/D box proteins; Nop1, Nop56, Nop58 and, being the primary binder, Snu13 (S5 Fig) [4]. We used the reads from previous CRAC studies of the core proteins Nop1, Nop56 and Nop58 [4,24] to map their precise binding sites on the 2D structure models of these snoRNPs (S5 Fig). Interestingly, these 2D models of snR4 and snR45 with the cross-linking sites of Kre33 along with core proteins, suggest that Kre33 and Nop1 presumably coexist on these snoRNPs–both enzymes interact at distinct sites. It is unlikely, however, that these snoRNAs mediate any conserved or specific 2′-*O*-methylation. Experimentally, it has been established that for efficient modification a minimal length of 7–8 base-pairs between a snoRNA-guide and its target is required and a maximum gap of 2 unpaired nucleotides between a functional guide and the associated D/D′ box is tolerated [3,4]. Only upstream of the D box in snR45 a conserved sequence is present that could direct modification of a short target sequence (5′-AAnUUUuU; nucleotide to be modified underlined), but in view of its length and irregularity, this seems a very poor guide for *2′-O*-methylation.

Our Kre33 CRAC analysis revealed three 18S rRNA binding sites: the 5′ domain, helix 34 and helix 45. Our data suggest that Kre33 predominantly interacts with the 5′ domain of 18S rRNA and our results support the hypothesis that its interaction with h34 and h45 is aided by snR4 and snR45, respectively. The Kre33 binding sites on 18S rRNA are spatially adjacent to that of snR4 and snR45 and a chimeric RNA that covers both GS2 of snR4 and its base-pairing site on h34 provides direct evidence.

Interestingly, Kre33 has been previously characterized as a component of the SSU processome and shown to physically interact with several components of the U3 snoRNP containing 90S particle including Rrp9, Enp1 and Nop14 [25,34]. In recent cryo-EM structures of the 90S particle from *Chaetomium thermophilum* and *S*. *cerevisiae*, Kre33 has been modeled into the head domain formed by the 5′ domain of 18S rRNA and suggested to bind there as a homodimer [23,34]. Our co-immunoprecipitation of U3 with Kre33 corroborated its association with the early 90S particle and our CRAC analysis precisely mapped Kre33 binding sites in the 5′ domain of 18S rRNA at nucleotide resolution, providing direct biochemical evidence.

Recent biochemical analyses of the early assembly of the SSU processome have shown that Kre33 joins the SSU processome relatively late once the 3′ major (h34) and minor (h45) domain of 18S rRNA have been transcribed [22]. Interestingly, within the 5′ domain Kre33 occupies the same region where early 90S assembly factors such as Efg1, Bfr2 and Lcp5 and snoRNAs snR44, snR49 and snR51 assemble [22,35]. The 90S particle undergoes several structural and compositional reorganizations during its transition from 90S to pre-40S, especially around the time Kre33 joins [35,36]. These transitions are aided by different helicases that facilitate the release of assembly factors including snoRNAs [37–39]. The stable base-pairing interactions between snoRNPs and the rRNA must be removed to advance ribosome synthesis. Any delay in the release of these snoRNPs results in substantial rRNA processing defects, as observed for many mutants of helicases involved in ribosome biogenesis [2]. Furthermore, since several snoRNA guided modifications cluster in functionally conserved regions of the ribosome, the modification machinery involved must be released to enable other enzymatic complexes to modify rRNA in the same region [36]. It is important to note that apart from its acetyltransferase domain, Kre33 has an N-terminal helicase domain, raising the intriguing possibility that Kre33 assists in the release of factors like Efg1, Bfr2 and Lcp5 or snoRNAs from the 5′ domain along with other helicases like Dbp4 and Has1 during the transition from 90S to 40S [36]. Our observation that mutations in the helicase domain of Kre33 cause early pre-rRNA processing defects, combined with aberrances in 18S-modification and association of several snoRNAs with the pre-ribosomes, strongly supports such a function for Kre33. It is equally possible that the snoRNA sedimentation defects observed in the K289A mutant are likely indirect, due to alternative folding of rRNA that precludes the accessibility/binding of these snoRNAs. On the other hand, as far as the effect on snR4 and snR45 is concerned, our RNA probing analyses revealed a significant change in DMS reactivity, prompting us to conclude that putative helicase activity of Kre33 is needed for annealing. Nevertheless, the impact in K289A mutant on snR4 and snR45 might also be a consequence of the pre-rRNA processing defect. This is further complicated by the possibility that putative helicase activity of Kre33 is directly involved in the removal of snR40 that remains attached to pre-ribosomal particles in the helicase mutant. There seem to be various subpopulations of 90S [23,34,35] and the observed changes in snoRNAs sedimentation might also relate to the 90S sub-population that relies on Kre33 activity.

The snoRNA-rRNA hybrids found in our CRAC-libraries yielded support for Kre33 action on snR4, snR40 and snR55 and indicated that Kre33 could regulate the interactions of 18S rRNA sequences with other snoRNAs, such as snR77, snR52, and possibly U14. CLASH on Kre33 should enable us to generate a more complete overview of snoRNA-rRNA interactions mediated by Kre33. Overall, we conclude that the defect of 18S rRNA processing in the helicase mutant is concomitant with or due to defective release of a subset of snoRNAs.

Apart from 18S rRNA acetylation in eukaryotes, Kre33 catalyzes ac^4^C-12 acetylation of serine and leucine tRNAs [18], which was confirmed by finding specific crosslinks of Kre33 with these tRNAs (S1A Fig). Neither snR4 nor snR45 influences acetylation of these tRNAs indicating that they are exclusively involved in the acetylation of 18S rRNA. Conversely, Tan1 is indispensable for Kre33-mediated tRNA acetylation and does not contribute to acetylation of rRNA. These data demonstrate that Kre33 utilizes different adaptor molecules to target different substrates in eukaryotes. Conspicuously, the human homolog of Kre33, NAT10, has been shown to exhibit lysine acetyltransferase activity especially towards microtubules and histones. In view of the very high sequence conservation between NAT10 and Kre33 it is tempting to speculate that Kre33 targets similar proteins in yeast. Future protein-protein interaction studies should be directed to identify other Kre33 adapter molecules. Another box C/D associated protein, the 2′-*O*-ribose methyltransferase Nop1/Fibrillarin, has been shown to target modification of histone H2A on glutamine residues both in yeast and human [40]. With the identification of different adaptor molecules of Kre33, it is now possible to uncouple acetylation of tRNA from that of rRNA, which provides a great opportunity to analyze the functional significance of each modification independently.

## Materials and methods

### Yeast strains and media

All yeast strains and plasmids used in the present study are listed in S2 Table. Yeast strains were grown at 30°C in YPD medium (1% w/v yeast extract, 2% w/v peptone, 2% w/v glucose) or in synthetic dropout (0.5% w/v ammonium sulphate, 0.17% w/v yeast nitrogen base, 2% w/v glucose). All kre33 mutant strains used in the present study were generated by distinctly transforming plasmids carrying Kre33 (pSH35), kre33-K289A (pSH35-a), kre33-H4545A (pSH35-c), kre33-R637A (pSH35-d) in a heterozygous deletion mutant of kre33 as constructed previously [18]. Tetrad analysis was performed to isolate a haploid kre33 deletion mutant containing respective complementing plasmids.

For growth analysis, yeast cells were grown over night in YPD medium and diluted to an OD_600nm_ of 1 followed by 1:10 serial dilutions. From the diluted cultures, 5 μl were spotted onto YPD plates and incubated at 37°C, 30°C or 16°C.

### Protein and RNA-Affinity purification

After an overnight growth in YPD medium, TAP or HTP tagged strains were diluted to an OD_600nm_ of 0.1 and were grown to an OD_600nm_ of 0.8 in 1 L YPD. Cells were collected by centrifugation and washed twice with ice-cold PBS. The cell pellet was resuspended in 1 volume of ice-cold TNM150 (50 mM Tris pH7.8. 1.5 mM MgCl_2_, 150 mM NaCl, 0.1% Igepal Ca-630 (NP-40), 5 mM β-mercaptoethanol) + protease inhibitors (Roche protease inhibitors cocktail tablets) and disrupted by shaking with 2.5 volumes of Zirconia beads (5x1 min with 1min resting on ice between each round). 3 volumes of ice-cool TMN-150 was then added to lysate which was clarified using centrifugation at maximum speed for 20 minutes at 4°C. The lysates were incubated for 2 h, at 4°C with end-over-end rotation with 125μL of IgG Sepharose beads. that had been equilibrated with TNM150 buffer (2 times washing with 3 mL of TNM150). The beads were washed 5 times with 1 mL of TNM150 (w/o protease inhibitors and were collected and resuspended in 250 μL of TNM150 (w/o protease inhibitors). For analysis of the protein, beads were directly boiled in protein loading buffer (4% SDS, 20% glycerol, 10% 2-mercaptoethanol, 0.004% bromophenol blue and 0.125 M Tris-HCl, pH approx. 6.8) and separated by SDS-PAGE and analyzed by Western blotting using anti-TAP antibody (Roche). RNA was isolated using phenol-chloroform extraction and the snoRNAs were characterized by Northern blotting as described previously [32]. Percent enrichment was calculated as the percent fold change in the signal of snoRNA band in total cell extract/input (T) versus pellet/eluate (P).

### CRAC analysis

CRAC was performed exactly as recently described [41]. Cells were cross-linked in the Vari-X-linker for 12 seconds and CRAC libraries were paired-end sequenced (50 bp) on a HiSeq2500 at Edinburgh Genomics, University of Edinburgh. The data reported in this paper have been deposited in the Gene Expression Omnibus (GEO) database, www.ncbi.nlm.nih.gov/geo (accession no. GSE87480). For CRAC data analysis, data from two biological replicates were analyzed as described[41]; yielding essentially the same outcomes; the data set with the better coverage (1,737,418 reads in data-set II vs 361,408 reads in data-set I) has been used for the presented figures. Hybrids of different RNAs crosslinked to Kre33 were identified in both data sets using the HYB-pipeline exactly as described [29]. As a control, both data sets were compared with the results of CRAC-experiments done with a variety of other RNA-binding proteins, which confirmed the specificity of the Kre33-crosslinks to 18S rRNA, tRNAs, snR4 and snR45 and the retrieved snoRNA-hybrids. Abundant snoRNAs that we routinely observe in our CRAC-data, such as U14 (snR128), U3 (snR17A, B) and snR190, were not specifically enriched in the Kre33 data-sets.

### Phylogenetic analysis and snoRNA model building

snoRNAs homologous to snR4 and snR45 were retrieved and aligned as described previously [4]. The C/D and D′/C′ motifs were identified by their conservation (S2 and S3 Figs) and secondary structure modeling was done on the premise that a similar structure would be formed by closely related RNA molecules and should rely on phylogenetic evidence, i.e. supported by compensatory base-changes in helices. Alignments were prepared with Jalview (www.jalview.org) and secondary structures generated with Varna (http://varna.lri.fr/). The base-pair interactions proposed to form the pseudoknot were identified by analyzing the alignments with SPuNC (http://www.ibi.vu.nl/programs/spuncwww/).

### 3D-structure modelling of Kre33

The 3D structure prediction was carried out with amino acid sequence of yeast Kre33 using recent protocol [42]. Kre33 structure was modelled on the crystal structure of its *E*. *coli* homolog, TmcA [29]. USCF Chimera was used to generate the ribbon-model for Kre33 [43].

### Mung bean nuclease assay and Reverse Phase High Performance Liquid Chromatography (RP-HPLC)

Mung bean nuclease protection assay and RP-HPLC analysis for acetylation were performed exactly as described before [18].

### Sucrose density gradient centrifugation for pre-ribosome and co-localization analysis

Sucrose gradient centrifugation for the pre-ribosome analysis and co-localization studies was performed as described previously [18].

### *In vivo* DMS (dimethyl sulfate) structure probing

A flask with 100 mL YPD media was inoculated with an overnight culture to a starting OD_600nm_ of 0.2 and grown at 30°C to an OD_600nm_ of 0.8 to 1.5. In the hood, two 15 mL aliquots of yeast culture in 50 mL polypropylene tubes were treated with 300 μL of 95% ethanol with 1:4, v/v DMS (Sigma) or without as a negative control and mixed vigorously for 15 seconds. Both aliquots were then incubated with shaking for 2 minutes at 30°C. The reaction was stopped by placing the tube on ice and adding 5 mL of 0.6 M β-mercaptoethanol and 5 mL of isoamyl alcohol. After addition of the stop solutions the tubes were vortexed for 15 seconds and then centrifuged at 3000x*g* at 4°C for 5 minutes. The cell pellets were washed with another 5 mL of 0.6 M β-mercaptoethanol.

Total RNA was isolated from the DMS treated cell pellet using GTC (4 M guanidium isothiocyanate, 50 mM Tris-HCl pH 8.0, 10 mM EDTA pH 8.0, 2% w/v sodium lauroyl sarcosinate and 150 mM β-mercaptoethanol) as described [18]. The sites of A and C methylation in snR4 and snR45 by DMS were mapped by primer extension using primers specific to snR4 (snR4-Mod-probe: TATTAATAGTTAAAGCACCG) and snR45 (snR45-Mod-probe: ATTTTATAAAAGCGTCCTTG). Primer extension was carried out exactly as described previously [7].

### Primer extension analysis for ribose methylation

Ribose methylation was analyzed by deoxynucleoside triphosphate (dNTP) concentration-dependent primer extension, exactly as described elsewhere [7]. Um898 in 25S rRNA was mapped with PE40_25S: TATCCTGAGGGAAACTTCGG, Um1269 and Gm1271 in 18S rRNA with PE-34_18S: TAAGGTCTCGTTCGTTATCGC. An rDNA sequence ladder was prepared to precisely map the location of the modifications.

## Supporting information

S1 Fig CRAC analysis revealed specific binding of Kre33 to leucine and serine tRNAs (A), 18S rRNA (B) and snoRNAs snR4 and snR45 (C). A) Overview of CRAC-results for Kre33 binding to serine and leucine tRNA species. The left y-axis shows the total number of times each nucleotide within an RNA fragment was mapped to the RNA sequence (x-axis); the right y-axis shows the number of reads carrying a substitution (red) or deletion (orange). Around the acetylated cytosine (*) a specific peak is observed in Kre33 cross-linked tRNAs. For other tRNA species or for the same species in control CRAC-experiments these substitution-peaks were not found. B) Read-alignments for Kre33 on the 18S rRNA show specific binding to the 5′ domain (pink), around helices 34 (orange) and 45 (blue). Putative crosslink sites (yellow circles) are seen as a gap in these sequences. Acetylated residues C1280 and C1773 (purple) fall within cross-linked segments. Note that Kre33 binding could be incompatible with that of modifying snoRNPs snR44, snR49, snR51, snR55, snR40, and snR83/Ruf3. C) As (B) but then for Kre33 binding to snR4 and snR45. Models of snoRNAs interacting with target rRNA sequence (blue) with the acetylated cytosine residue (red) are as in Fig 4.
(PDF)

S2 Fig Phylogenetic analysis of snR45. A) Alignment of snoRNAs homologous to snR45. Sequences were retrieved and aligned as described previously [4]. The guide sequences (GS1 and GS2) and the C/D and C′ motifs stand out due to their high level of conservation (deep blue shade). The non-canonical D′ motif is quite variable and assigned based on 2D-modelling (B). Dot-bracket-notation shows the general 2D-structure (top-line). B) Secondary structure models of snR45 for the indicated yeast species. Shown are the conserved regions with guide sequences GS1 and GS2 (red), the C/D and C′/D′ motifs (black), the pseudo-knot (olive) and helices with strong phylogenetic support (teal).
(PDF)

S3 Fig Phylogenetic analysis of snR4 with alignment (A) and 2D-models (B) as described for S2 Fig. Note, *Torulaspora delbrueckii* snR45 could be modeled with a D′ box using a canonical sequence (CUGA, red ellipse) instead of the CUGU found in *S*. *cerevisiae* and closely related species.
(PDF)

S4 Fig snR4 is involved in 18S rRNA acetylation at C1280. A) Overlaid RP-HPLC chromatograms of the nucleosides derived from fragments isolated using mung bean nuclease assay, containing ac^4^C1280 (oligo 34, black) and ac^4^C1773 (oligo 45, red) isolated from a strain lacking snR4 (Δsnr4). Loss of snR4 leads to complete loss of ac^4^C1280 without affecting ac^4^C1773. B) Overlaid RP-HPLC chromatogram of tRNAs from strains lacking snR4 *(*Δsnr4, green), snR45 (Δsnr45, blue) and Tan1 (Δtan1, red) strain. Loss of snR4 and snR45 does not affect tRNA acetylation.
(PDF)

S5 Fig Crosslinking of Nop56, Nop58 and Nop1 to snR4 and snR45. 2D structure models (top panel) based on phylogenetic comparison and DMS-modification for snR4 (A) and snR45 (B) showing Kre33 cross-linked sites (identified in the present study, yellow ovals), along with the binding regions (transparent) and cross-linking sites (opaque ovals) of Nop1 (purple), Nop56 (green), and Nop58 (blue) determined previously [4] and as colored squares overlaid on the alignments (bottom panel) of unique reads with the snoRNA sequences. For further details, see legend Fig 4.
(PDF)

S6 Fig Physical interaction of snR4 and snR45 with 18S rRNA is indispensable for 18S rRNA acetylation. 2D models of snR4 (A) and snR45 (B) along with zoomed-in views (C and D) for mutated regions. The dotted red box in (B) indicate a mutation that interfered with stable expression of the snoRNA. See for further details the legend for Fig 5, F) RP-HPLC chromatograms of 18S rRNA isolated from a strain carrying a double deletion for *SNR4* and *SNR45* expressing the indicated mutant versions of snR4 (E) and snR45 (F). (G) Northern blot developed with snoRNA-specific probes showing stable expression of snr4 and snr45 mutants. 5S rRNA was used as loading control. H) Sedimentation profile of snR4 (snr4-b) and snR45 (snr45-b) with an altered GS2 (cf. Fig 5) to test their association with ribosomes/pre-ribosomes; northern analysis in (H), snoRNA-specific probes and western-blots were probed with anti-TAP, anti-Nop1, and anti-Nop2 antibodies to detect Kre33-TAP, Nop1 and Nop2, respectively.
(PDF)

S7 Fig Sedimentation profiles of snR4 and snR45 upon Kre33 depletion. Kre33 was depleted using a strain containing HA tagged Kre33 under galactose promoter (pGAL1::3HA-kre33) that was grown in YPGSR (yeast extract, peptone, galactose–sucrose–raffinose, 2% w/v each) to mid-log phase, washed in pre-warmed water and transferred to YPD for up to 12 h. We compared t0 (time point at which cells were transferred to YPD) and t6 (6hours after transfer to YPD). A) Western blot showing that glucose mediated depletion of Kre33 (top panel) was extremely effective and after 6 hours Kre33 was depleted to a level beyond the Western blot detection limit. The bot was developed using mouse anti-HA (SIGMA) and goat anti-mouse-HRP (Santa Cruz Biotechnology) antibodies. Glucose 6 phosphate dehydrogenase (G6PDH) was used as a loading control (lower panel). The blot was developed using rabbit anti G6PDH and donkey-anti rabbit (Santa Cruz Biotechnology) antibodies. B) Sedimentation profiles of snR4 and snR45 at t0 and t6.
(PDF)

S1 Table Hybrids containing snoRNA sequences. (PDF)

S2 Table Strains, plasmids and oligonucleotides. (PDF)
