# Supplementary figures and images for: Specialized box C/D snoRNPs act as antisense guides to target RNA base acetylation
Source: PLoS Genet. 2017 May 24;13(5):e1006804. doi: 10.1371/journal.pgen.1006804 (PMC5464676; doi:10.1371/journal.pgen.1006804)

A

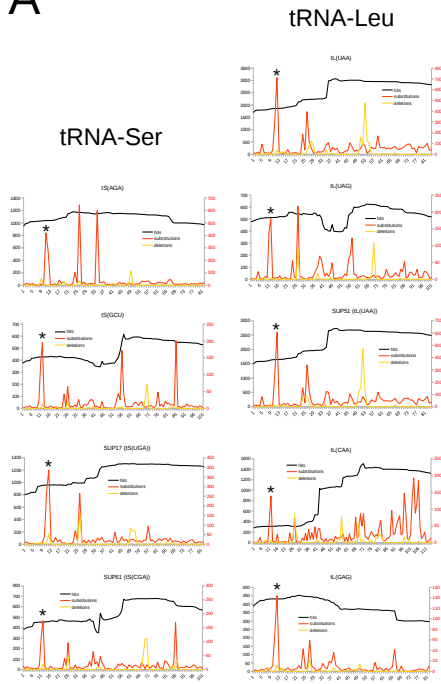

B

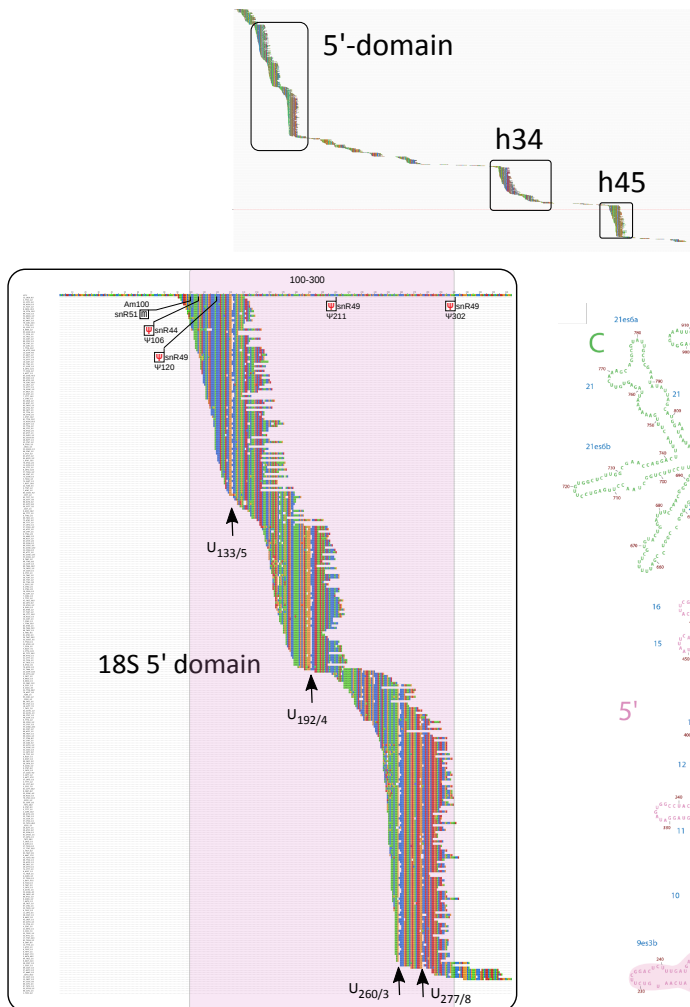

C

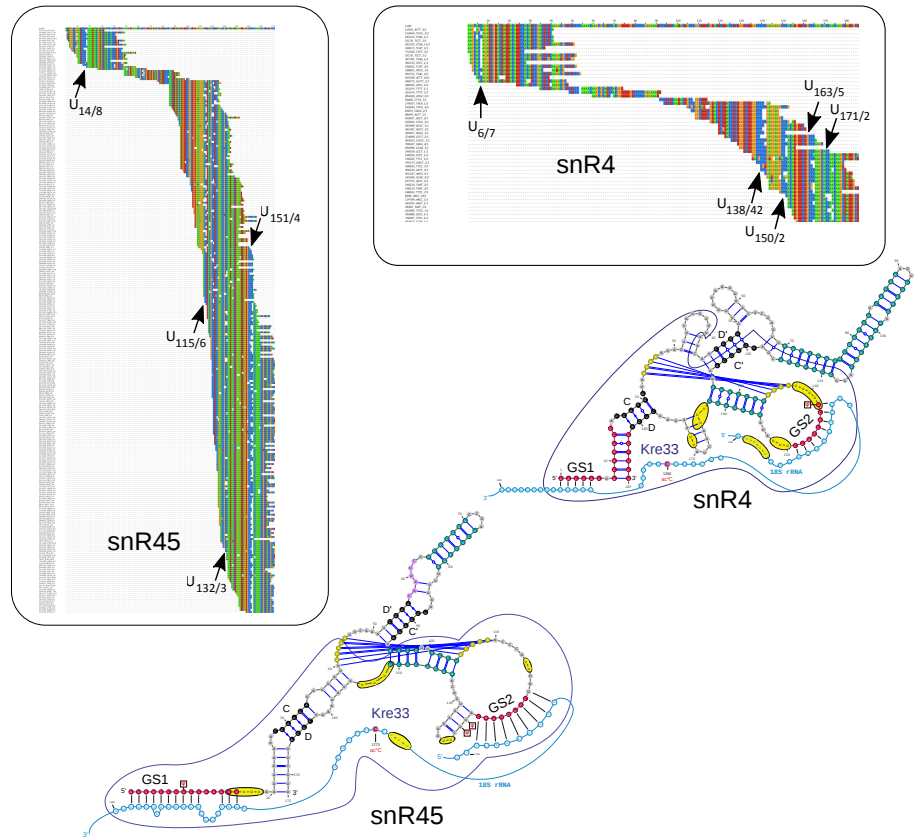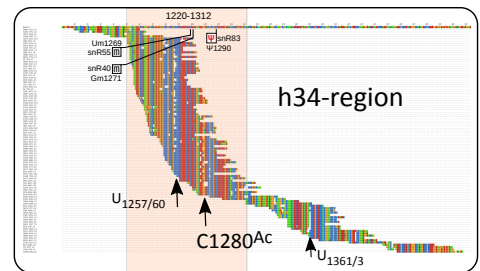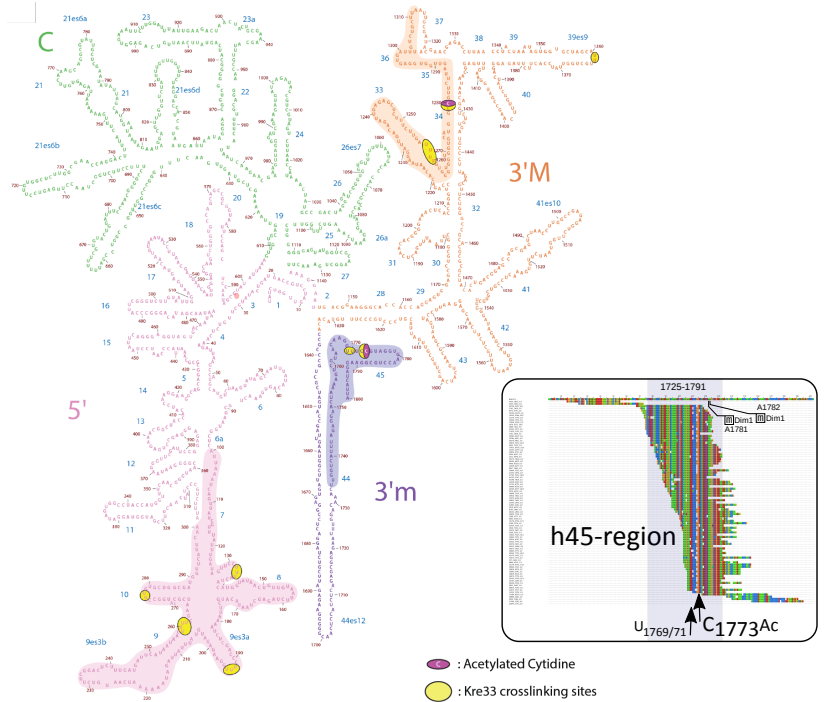

Supplement: S1 Fig — CRAC analysis revealed specific binding of Kre33 to leucine and serine tRNAs (A), 18S rRNA (B) and snoRNAs snR4 and snR45 (C). A) Overview of CRAC-results for Kre33 binding to serine and leucine tRNA species. The left y-axis shows the total number of times each nucleotide within an RNA fragment was mapped to the RNA sequence (x-axis); the right y-axis shows the number of reads carrying a substitution (red) or deletion (orange). Around the acetylated cytosine (*) a specific peak is observed in Kre33 cross-linked tRNAs. For other tRNA species or for the same species in control CRAC-experiments these substitution-peaks were not found. B) Read-alignments for Kre33 on the 18S rRNA show specific binding to the 5′ domain (pink), around helices 34 (orange) and 45 (blue). Putative crosslink sites (yellow circles) are seen as a gap in these sequences. Acetylated residues C1280 and C1773 (purple) fall within cross-linked segments. Note that Kre33 binding could be incompatible with that of modifying snoRNPs snR44, snR49, snR51, snR55, snR40, and snR83/Ruf3. C) As (B) but then for Kre33 binding to snR4 and snR45. Models of snoRNAs interacting with target rRNA sequence (blue) with the acetylated cytosine residue (red) are as in Fig 4. (PDF) [file pgen.1006804.s001.pdf]

snR45

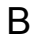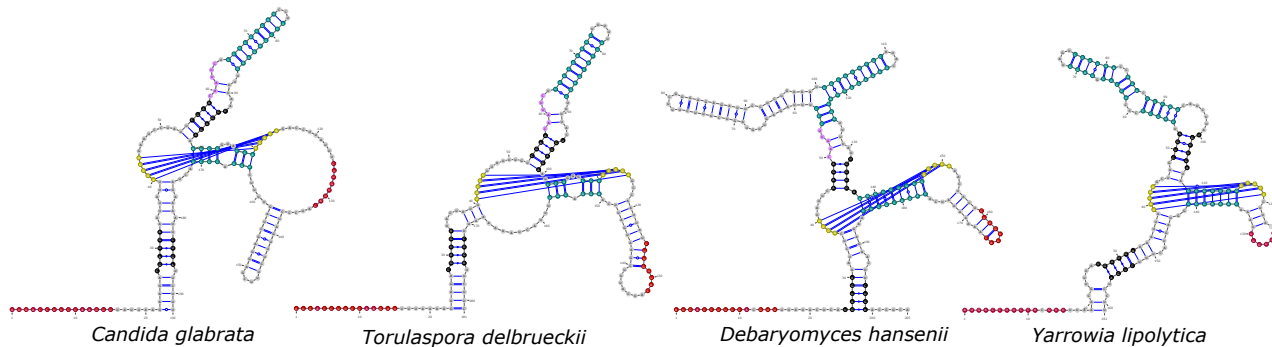

Supplement: S2 Fig — A) Alignment of snoRNAs homologous to snR45. Sequences were retrieved and aligned as described previously [4]. The guide sequences (GS1 and GS2) and the C/D and C′ motifs stand out due to their high level of conservation (deep blue shade). The non-canonical D′ motif is quite variable and assigned based on 2D-modelling (B). Dot-bracket-notation shows the general 2D-structure (top-line). B) Secondary structure models of snR45 for the indicated yeast species. Shown are the conserved regions with guide sequences GS1 and GS2 (red), the C/D and C′/D′ motifs (black), the pseudo-knot (olive) and helices with strong phylogenetic support (teal). (PDF) [file pgen.1006804.s002.pdf]

**A**

# Mung Bean Nuclease Assay

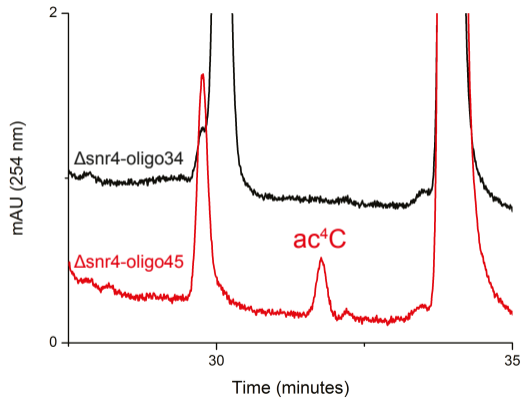**B**

# RP-HPLC (tRNA)

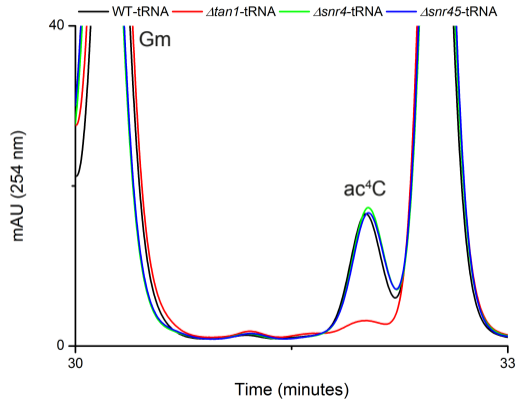

Supplement: S4 Fig — A) Overlaid RP-HPLC chromatograms of the nucleosides derived from fragments isolated using mung bean nuclease assay, containing ac4C1280 (oligo 34, black) and ac4C1773 (oligo 45, red) isolated from a strain lacking snR4 (Δsnr4). Loss of snR4 leads to complete loss of ac4C1280 without affecting ac4C1773. B) Overlaid RP-HPLC chromatogram of tRNAs from strains lacking snR4 (Δsnr4, green), snR45 (Δsnr45, blue) and Tan1 (Δtan1, red) strain. Loss of snR4 and snR45 does not affect tRNA acetylation. (PDF) [file pgen.1006804.s004.pdf]

A

snR4

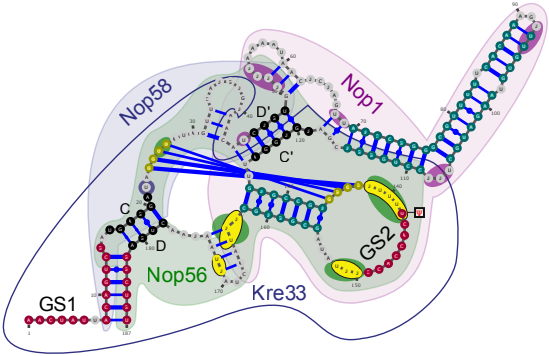

B

snR45

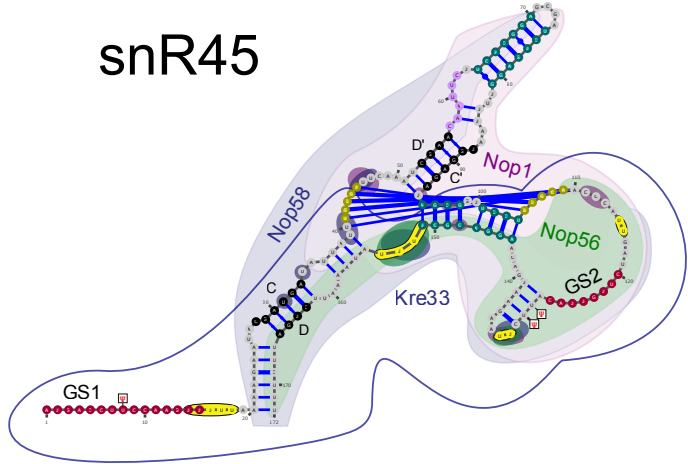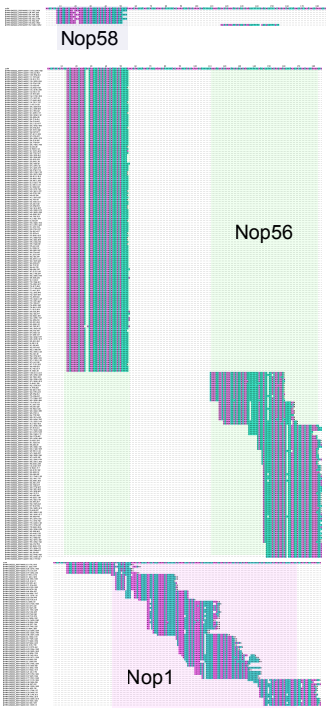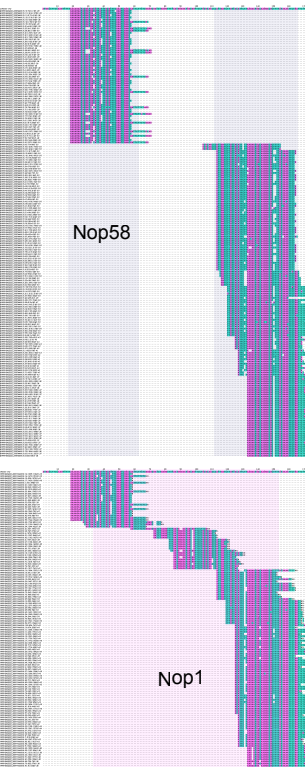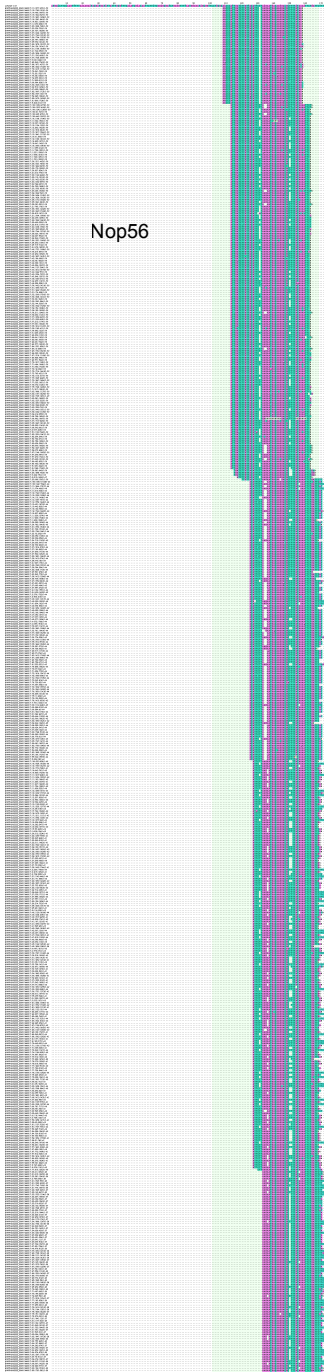

Supplement: S5 Fig — 2D structure models (top panel) based on phylogenetic comparison and DMS-modification for snR4 (A) and snR45 (B) showing Kre33 cross-linked sites (identified in the present study, yellow ovals), along with the binding regions (transparent) and cross-linking sites (opaque ovals) of Nop1 (purple), Nop56 (green), and Nop58 (blue) determined previously [4] and as colored squares overlaid on the alignments (bottom panel) of unique reads with the snoRNA sequences. For further details, see legend Fig 4. (PDF) [file pgen.1006804.s005.pdf]

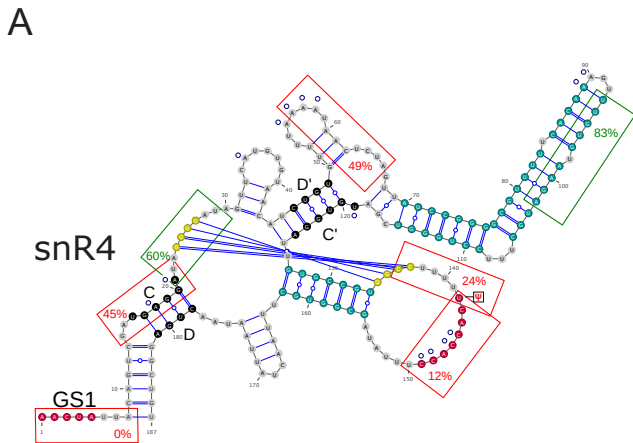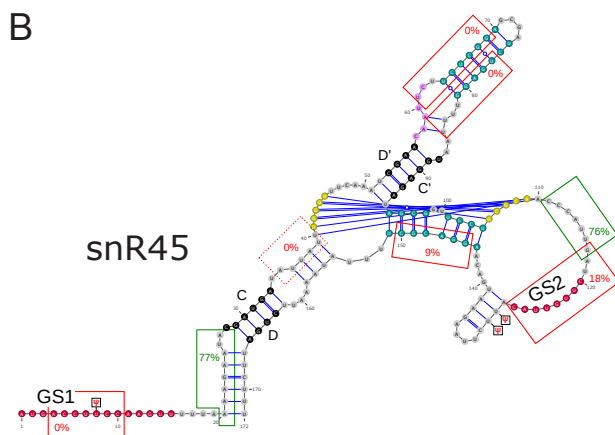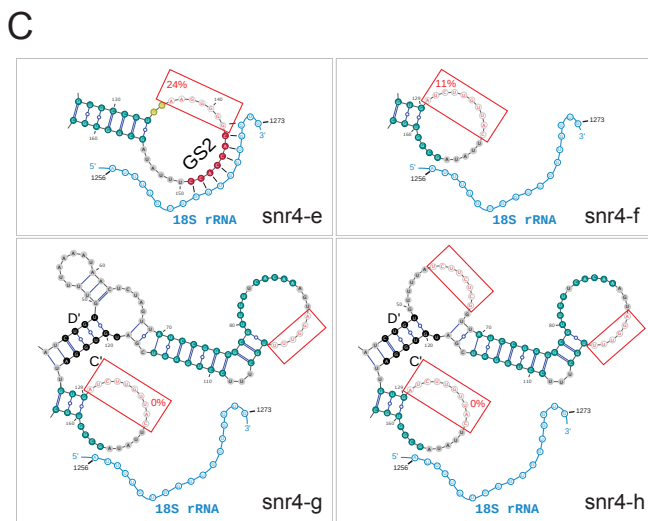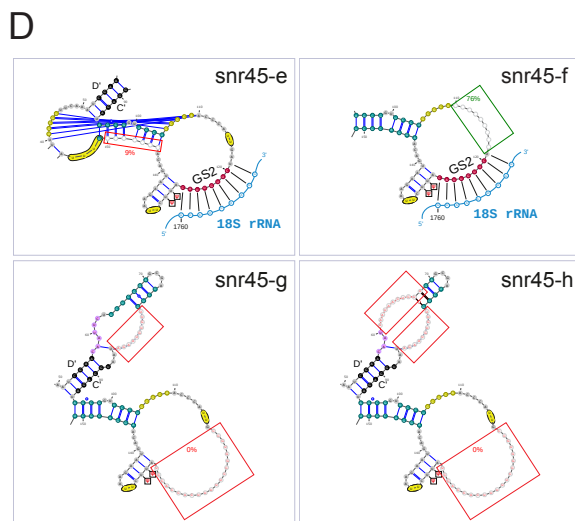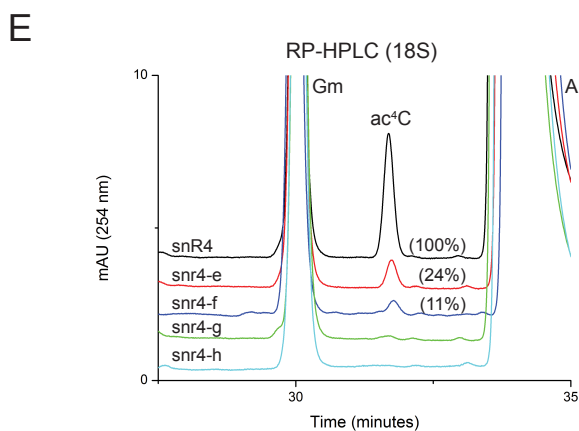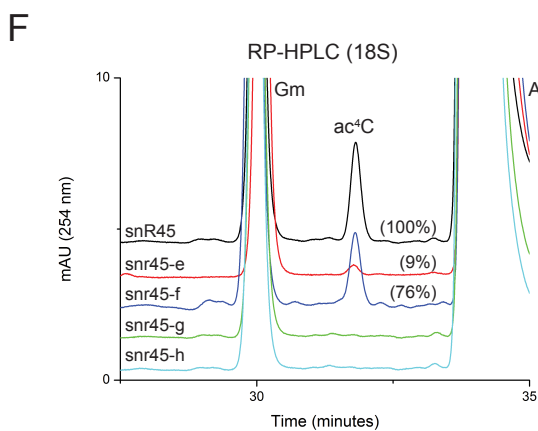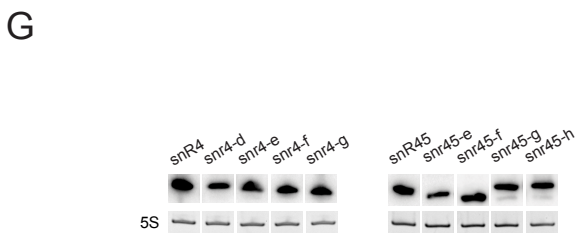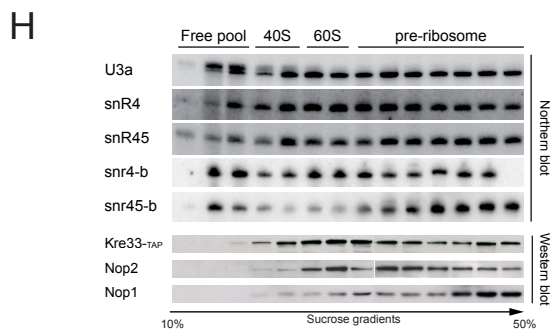

Supplement: S6 Fig — 2D models of snR4 (A) and snR45 (B) along with zoomed-in views (C and D) for mutated regions. The dotted red box in (B) indicate a mutation that interfered with stable expression of the snoRNA. See for further details the legend for Fig 5, F) RP-HPLC chromatograms of 18S rRNA isolated from a strain carrying a double deletion for SNR4 and SNR45 expressing the indicated mutant versions of snR4 (E) and snR45 (F). (G) Northern blot developed with snoRNA-specific probes showing stable expression of snr4 and snr45 mutants. 5S rRNA was used as loading control. H) Sedimentation profile of snR4 (snr4-b) and snR45 (snr45-b) with an altered GS2 (cf. Fig 5) to test their association with ribosomes/pre-ribosomes; northern analysis in (H), snoRNA-specific probes and western-blots were probed with anti-TAP, anti-Nop1, and anti-Nop2 antibodies to detect Kre33-TAP, Nop1 and Nop2, respectively. (PDF) [file pgen.1006804.s006.pdf]

A

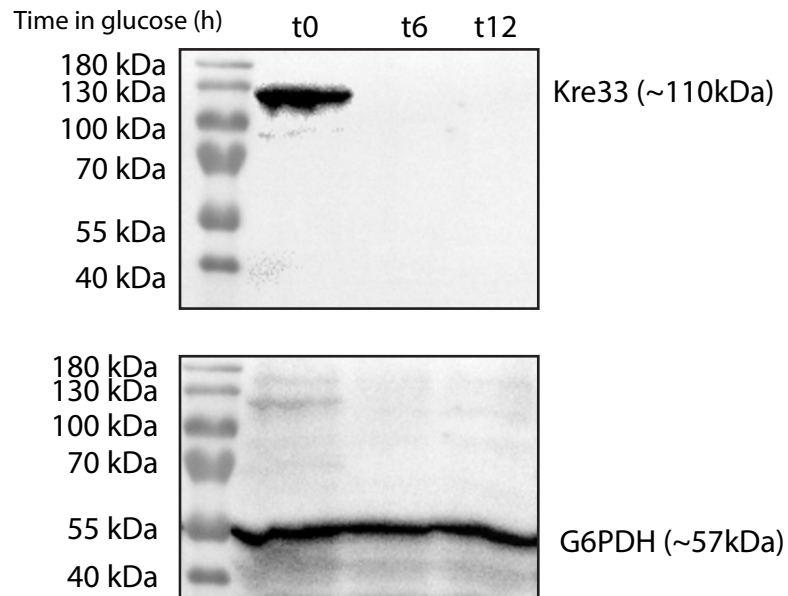

B

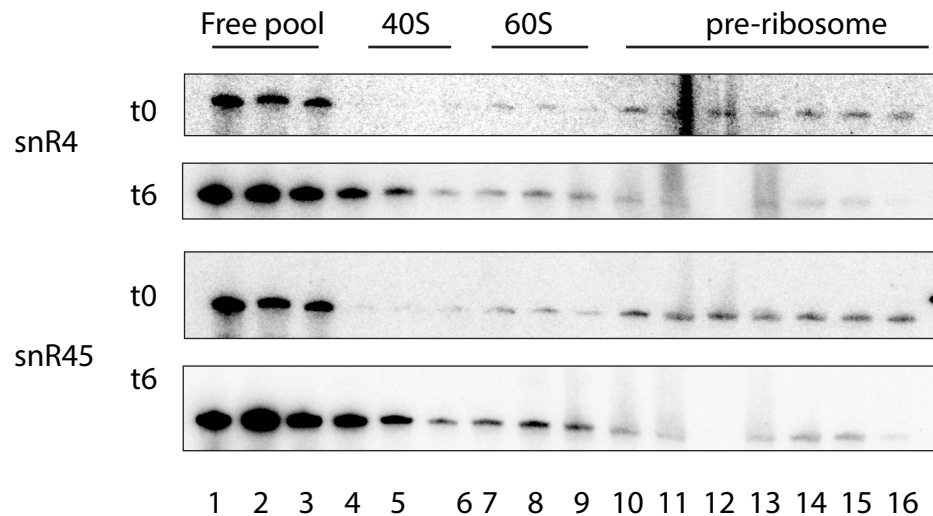

Supplement: S7 Fig — Kre33 was depleted using a strain containing HA tagged Kre33 under galactose promoter (pGAL1::3HA-kre33) that was grown in YPGSR (yeast extract, peptone, galactose–sucrose–raffinose, 2% w/v each) to mid-log phase, washed in pre-warmed water and transferred to YPD for up to 12 h. We compared t0 (time point at which cells were transferred to YPD) and t6 (6hours after transfer to YPD). A) Western blot showing that glucose mediated depletion of Kre33 (top panel) was extremely effective and after 6 hours Kre33 was depleted to a level beyond the Western blot detection limit. The bot was developed using mouse anti-HA (SIGMA) and goat anti-mouse-HRP (Santa Cruz Biotechnology) antibodies. Glucose 6 phosphate dehydrogenase (G6PDH) was used as a loading control (lower panel). The blot was developed using rabbit anti G6PDH and donkey-anti rabbit (Santa Cruz Biotechnology) antibodies. B) Sedimentation profiles of snR4 and snR45 at t0 and t6. (PDF) [file pgen.1006804.s007.pdf]
