# Supplementary material for: Specialized box C/D snoRNPs act as antisense guides to target RNA base acetylation
Source: PLoS Genet. 2017 May 24;13(5):e1006804. doi: 10.1371/journal.pgen.1006804 (PMC5464676; doi:10.1371/journal.pgen.1006804)
Supplement: S3 Fig — Phylogenetic analysis of snR4 with alignment (A) and 2D-models (B) as described for S2 Fig. Note, Torulaspora delbrueckii snR45 could be modeled with a D′ box using a canonical sequence (CUGA, red ellipse) instead of the CUGU found in S. cerevisiae and closely related species. (PDF) [file pgen.1006804.s003.pdf]

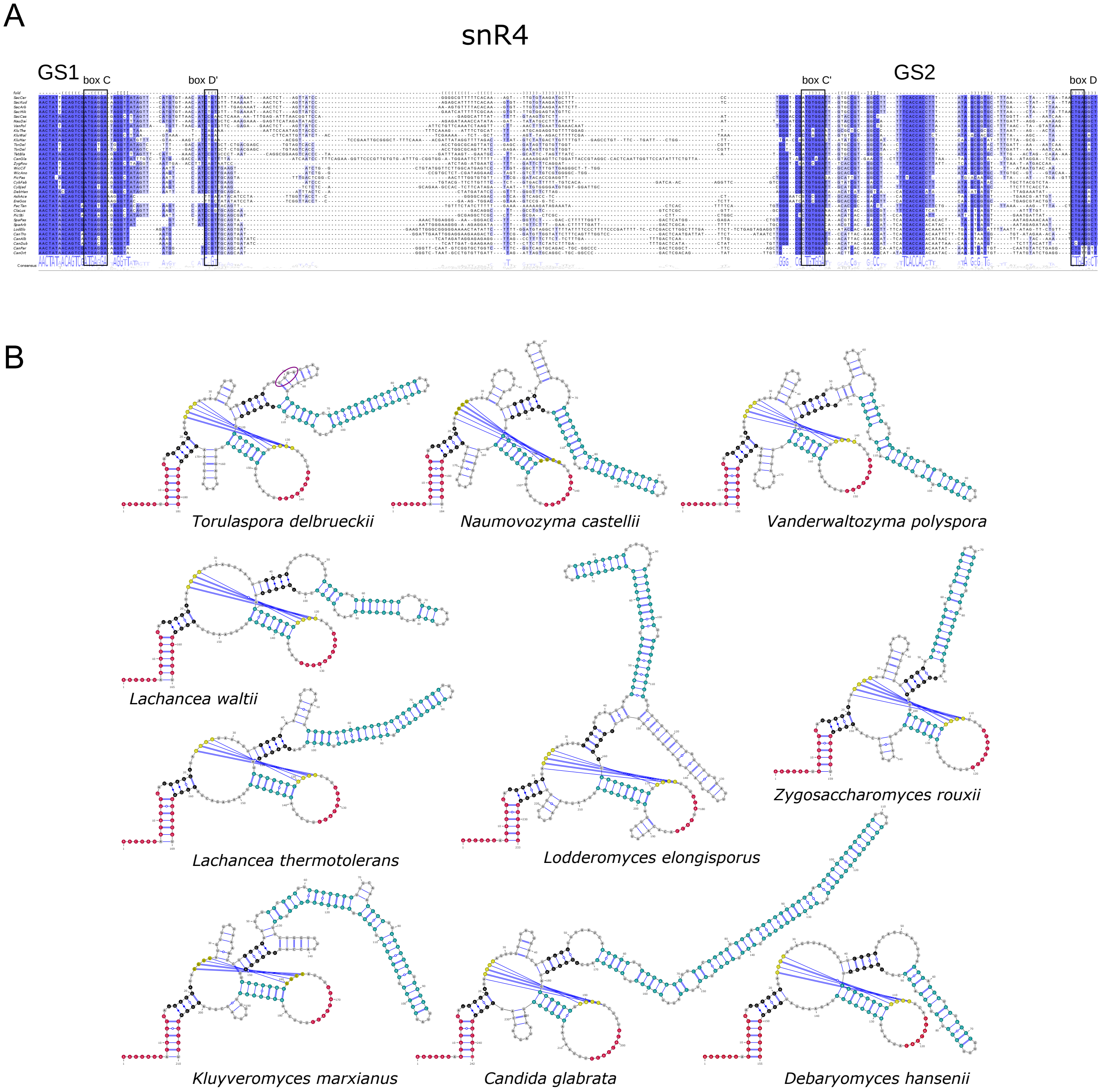
A
snR4
GS1
box C
box D'
box C'
GS2
box D
B
Torulaspora delbrueckii
Naumovozyma castellii
Vanderwaltozyma polyspora
Lachancea waltii
Lachancea thermotolerans
Lodderomyces elongisporus
Zygosaccharomyces rouxii
Kluyveromyces marxianus
Candida glabrata
Debaryomyces hansenii
