# Supplementary material for: Specialized box C/D snoRNPs act as antisense guides to target RNA base acetylation
Source: PLoS Genet. 2017 May 24;13(5):e1006804. doi: 10.1371/journal.pgen.1006804 (PMC5464676; doi:10.1371/journal.pgen.1006804)
Supplement: S2 Table — (PDF) [file pgen.1006804.s009.pdf]

S2 Table. Strains, plasmids and oligonucleotides

Name	Description	Source
CEN.PK1303-5A	MAT a, ura3-52;his3-Δ1; leu2-3,112; trp1-289; MAL2-8^c , SUC2, snr45(1,172)::loxP-kanMX4-loxP	This study
CEN.PK1351-5B	MAT a, ura3-52;his3-Δ1; leu2-3,112; trp1-289; MAL2-8^c , SUC2, snr4(-50,186)::loxP-kanMX4-loxP	This study
CEN.WA4-9C	MAT α, ura3-52;his3-Δ1; leu2-3,112; trp1-289; MAL2-8^c , SUC2, snr4(-50,186)::loxP-kanMX4-loxP; snr45(1,172)::loxP-kanMX4-loxP	This study
BY4741	MATa; his3Δ1; leu2Δ0; met15Δ0; ura3Δ0	Euroscarf
SC1403	MATa; ura3-52; leu2-3,112; KRE33::TAP-KlURA3	Euroscarf
SC1541	MATa; ura3-52; leu2-3,112; NOP58::TAP-KlURA3	Euroscarf
SC1110	MATa; ura3-52; leu2-3,112; GAR1::TAP-KlURA3	Euroscarf
BY.WA5-2B	MAT α, ura3Δ0; leu2Δ0; his3Δ1; KRE33::kanMX4 + pSH35	(Sharma et al., 2015)
BY.WA6-1B	MAT α, ura3Δ0; leu2Δ0; his3Δ1; KRE33::kanMX4 + pSH35a	(Sharma et al., 2015)
BY.WA8-1B	MAT α, ura3Δ0; leu2Δ0; his3Δ1; KRE33::kanMX4 + pSH35c	(Sharma et al., 2015)
BY.WA9-1C	MAT A, ura3Δ0; leu2Δ0; his3Δ1; KRE33::kanMX4 + pSH35d	(Sharma et al., 2015)
pJN23	A derivative pRS426 plasmid carrying *SNR4*	This study
pJN23a	A derivative pRS426 plasmid carrying snr4(1,8)::TTGATTAT	This study
pJN23b	A derivative pRS426 plasmid carrying snr4(142,150)::GGTGGTGGG	This study
pJN23c	A derivative pRS426 plasmid carrying snr4(93,102)::GGTTGATTCT	This study
pJN23d	A derivative pRS426 plasmid carrying snr4(56,66)::TTTAATGAGGT	This study
pJN23e	A derivative pRS426 plasmid carrying snr4(136,142)::AAGGGGG	This study
pJN23f	A derivative pRS426 plasmid carrying snr4(130,150)::ATCTTGTTAC	This study
pJN23g	A derivative pRS426 plasmid carrying snr4(93,102)::AGGTTT; (130,150)::ATCTTGTTAC	This study
pJN23h	A derivative pRS426 plasmid carrying snr4(56,66)::TCTTCTCG; (93,102)::AGGTTT; (130-150)::ATCTTGTTAC	This study
pJN28	A derivative pRS426 plasmid carrying *SNR45*	This study
pJN28a	A derivative pRS426 plasmid carrying snr45(3,10)::GACCTTCC	This study
pJN28b	A derivative pRS426 plasmid carrying snr45(77,84)::GTAACCAG	This study
pJN28c	A derivative pRS426 plasmid carrying snr45(61,68)::AGGACGGC	This study
pJN28d	A derivative pRS426 plasmid carrying snr45(117,127)::TTGGAACAATG	This study
pJN28e	A derivative pRS426 plasmid carrying snr45(144,150)::Δ	This study
pJN28f	A derivative pRS426 plasmid carrying snr45(110,118)::Δ	This study
pJN28g	A derivative pRS426 plasmid carrying snr45(79,84)::TTGTGTAAGA; (118,127)::CCGTGGCCTTTTTCACCACCT	This study
pJN28h	A derivative pRS426 plasmid carrying snr45(61,68)::AAATAACTCTA; (79,84)::TTGTGTAAGA; (118,127)::CCGTGGCCTTTTTCACCACCT	This study
pSH35	A derivative pPK468 plasmid carrying Kre33-7xHis	(Sharma et al., 2015)
pSH35a	A derivative pPK468 plasmid carrying kre33-K289A	(Sharma et al., 2015)
pSH35c	A derivative pPK468 plasmid carrying kre33-H545A	(Sharma et al., 2015)
pSH35d	A derivative pPK468 plasmid carrying kre33-R637A	(Sharma et al., 2015)
**Name**	**Sequence 5′ —> 3′**	
pJN23-A1	GAATTGGGTACCGGGCCCCCCCTCGAGGTCGACGGTATCGATAAGAAAG CTTTGTAAATATG	
pJN23-A2	GAGCTCCACCGCGGTGGCGGCCGCTCTAGAACTAGTGGATCGGTATAAT AGAAGAATTTTAG	

pJN28-A1	GAATTGGGTACCGGGCCCCCCCTCGAGGTCGACGGTATCGATAGATTAACTTGATAATGGAGTTG	
pJN28-A2	GAGCTCCACCGCGGTGGCGGCCGCTCTAGAACTAGTGGATCGAGACCAATCGACAGTCTTC	
snR4-nb	CTAGAGTTATTTTAAAACAC	
snR45-nb	GCGCAGGAACCGCTATCTCC	
snR4-Mod-probe	TATTAATAGTTAAAGCACCG	
snR45-Mod-probe	ATTTTATAAAAGCGTCCTTG	
pJN23a-sp-FP	CATTTTTTTATTTCAAAATCCCCATCTTGATAATCAGTCGATGAGGATAGGTTATAG	
pJN23a-sp-RP	CTATAACCTATCCTCATCGACTGATTATCAAGATGGGGATTTTGAAATAAAAAAATG	
pJN23b-sp-F2	GATTGTGCCGTGGCCTTTTGGTGGTGGGTTATAGCGGTGCTTTAACTATTAATAACTG	
pJN23b-sp-R2	ATTAATAGTTAAAGCACCGCTATAACCCACCACCAAAAGGCCACGGCACAATCCACATC	
snR4-cF	GGCGTTTTTCACAAAGTGGTTGATTCTTGCTTTCCTGGGTCGATGTGG	
snR4-cR	CGACCCAGGAAAGCAAGAATCAACCACTTTGTGAAAAACGCCCC	
pJN23d-sp-F2	GTGTAACATCTGTGTTTTATTTAATGAGGTGTTATCCGGGGCGTTTTTCACAAAG	
pJN23d-sp-R2	GTGAAAAACGCCCCGGATAACACCTCATTAAATAAAACACAGATGTTACACATG	
snR4-eF	GGGTCGATGTGGATTGTGCCGTGGAAGGGGGCACCACCTTTATAGCGGTGCTTTAAC	
snR4-eR	GCACCGCTATAAAGGTGGTGCCCCCTTCCACGGCACAATCCACATCGACCCAGGAAAGC	
snR4-fF	GGATTGTGATCTTGTTACTTATAGCGGTGCTTTAACTATTAATAAC	
snR4-fR	CGCTATAAGTAACAAGATCACAATCCACATCGACCCAGG	
snR4-gF2	CACAAAGTAGGTTTTGCTTTCCTGGGTCGATGTG	
snR4-gR2	GCAAAACCTACTTTGTGAAAAACGCCCCG	
pJN23h-sp-F3	CATCTGTGTTTTATCTTCTCGGTTATCCGGGGCGTTTTTCACAAAGTAGGTTTTGC	
pJN23h-sp-R3	CCCGGATAACCGAGAAGATAAAACACAGATGTTACACATGAACTATAACC	
pJN28a-sp-FP	GGTTTCATTTAAAGAAACCAATCTGGAAGGAAGTTTTTAAAAGAATACGATG	
pJN28a-sp-RP	CGTATTCTTTTAAAAACTTCCTTCCAGATTGGTTTCTTTAAATGAAACCTCC	
pJN28c-sp-F2	GCGTTTCAAATCGAACAATAGGACGGCGAGCGATCTGAGGTTTTAATGGAG	
pJN28c-sp-R2	CATTAAAACCTCAGATCGCTCGCCGTCCTATTGTTCGATTTGAAACGCAAATAATATC	
pJN28e-sp-F2	CTTGTTACATTCTTAAGAATGACATTTTATAAAATTCTGATTCTTTTTTCTTTC	
pJN28e-sp-R2	GAAAGAAAAAAGAATCAGAATTTTATAAAATGTCATTCTTAAGAATGTAACAAGATC	
snR45-1	CTTGGAAGGTCATTGGTTTCTTTAAATGAAACCTCCAAAGAAAATCTAACGCGTCTTGGTAAAATATATGTACCGAGTGTTTCGCTTTTTATACC	
snR45-2	GGAGGTTTCATTTAAAGAAACCAATGACCTTCCAAGTTTTTAAAAGAATACGATGATATTATTTGCGTTTCAAATCGAACAATTCTTCTCGGAGCGATCTGAGG	
snR45-2b	GGAGGTTTCATTTAAAGAAACCAATGACCTTCCAAGTTTTTAAAAGAATACGATGATATTATTTGCGTTTCAAATCGAACAATTCTTCTCGGAGCGATC	

snR45-3b	GTCCTTGTCATTCTTAAGAATGTAACAAGATCAATGGGTTGCGCAGGAACCGCTATCTCCATTACTGGTTACGATCGCTCCGAGAAGAATTGTTCGATTTGAAACGC	
snR45-4b	CCCATTGATCTTGTTACATTCTTAAGAATGACAAGGACGCTTTTATAAAATTCTGATTCTTTTTTCTTTCATTCTTTTGTTGTTTTTGACTTGGGAG	
snR45-3d	CCTTGTCATTCTTAAGAATCATTGTTCCAAAATGGGTTGCGCAGGAACCGCTATCTCCATTAAAACCTCAGATCGCTCCGAGAAGAATTGTTCGATTTG	
snR45-4d	GCAACCCATTTTGGAACAATGATTCTTAAGAATGACAAGGACGCTTTTATAAAATTCTGATTCTTTTTTCTTTCATTCTTTTGTTGTTTTTGACTTGGG	
snR45-3f	GCGTCCTTGTCATTCTTAAGAATGTAACAAGATGCGCAGGAACCGCTATCTCCATTAAAACCTCAGATCGCTCCGAGAAGAATTGTTCGATTTG	
snR45-4f	GGTTCCTGCGCATCTTGTTACATTCTTAAGAATGACAAGGACGCTTTTATAAAATTCTGATTCTTTTTTCTTTCATTCTTTTGTTGTTTTTGACTTGGG	
snR45-3g	GAATAGGTGGTGAAAAAGGCCACGGCAATGGGTTGCGCAGGAACCGCTATCTCCATTATCTTACACAACAGATCGCTCCGAGAAGAATTGTTCGATTTG	
snR45-2h	CATTTAAAGAAACCAATGACCTTCCAAGTTTTTAAAAGAATACGATGATATTATTTGCGTTTCAAATCGAACAATAAATAACTCTAGAGCGATCTGTTG	
snR45-3h	GAATAGGTGGTGAAAAAGGCCACGGCAATGGGTTGCGCAGGAACCGCTATCTCCATTATCTTACACAACAGATCGCTCTAGAGTTATTTATTGTTCG	
snR45-4h	CCCATTGCCGTGGCCTTTTTCACCACCTATTCTTAAGAATGACAAGGACGCTTTTATAAAATTCTGATTCTTTTTTCTTTCATTCTTTTGTTGTTTTTGAC	
snR45-5	CTAGTCTACAATTTAAGAACATATAAAAAGCTACTTGTGTACAGATGAGATGACTACTCCCAAGTCAAAAACAACAAAAGAATGAAAGAAAAAAG	
probe 33	CGCTGCTCACCAATGG	
probe 20	TGAGAAGGAAATGACGCT	
probe 9	CCCACCTATTCCCTCTTGC	
probe 5	GATTGCTCGAATGCCCAAAG	
probe 4	CGGTTTTAATTGTCCTA	
PE_40_25S	TATCCTGAGGGAAACTTCGG	
PE_34_18S	TAAGGTCTCGTTCGTTATCGC	
