# Supplementary material for: Specialized box C/D snoRNPs act as antisense guides to target RNA base acetylation
Source: PLoS Genet. 2017 May 24;13(5):e1006804. doi: 10.1371/journal.pgen.1006804 (PMC5464676; doi:10.1371/journal.pgen.1006804)
Supplement: S1 Table — (PDF) [file pgen.1006804.s008.pdf]

S1 Table. Hybrids containing snoRNA sequences

Read No. ‡	Sequence of hybrids between 18S rRNA and snoRNA	ΔG*	5' Source	from	to	3' Source (motif)	from	to
130876-1_2	TAGTGAAACTGCGAATGGCTCCACGGTGATGAAAGACTGGT	-3	RDN37-1	80	99	U14 / snR128 (C)	2	21
-12350-2_5	AGTGAAACTGCGAATGGCTCAACATTCGCAGTTTCCACGG	-20.6	RDN37-2	80	100	U14 / snR128	27	46
63756-1_6	TTGGCCTTTTTCACCACCTTTAT***TTTT***GTGGGTGGTGGTGC	-14.5	snR4	133	154	RDN37-1	1257	1274
97648-1_3	TAAGGAAGGCAGCAGGCGCGCTACTATGATGAATGACATTA	-1.8	RDN37-1	416	435	snR52 (C)	1	20
99477-1_3	TGGAGGGCAAGTCTGGTGCCAGCAAAAAGCTGTGCACCAGT	-18.1	RDN37-1	547	570	snR40	14	31
133303-1_2	TGGTGCATGGCCGTTCTTGGTAAATGACGAGAAAAAAGCTG	-7.4	RDN37-1	1269	1289	snR40 (C)	1	22
301878-1_1	TGGTGGTGCATGGCCGTTCTTGTAAATGACGAGAAAAAAGC	-3.2	RDN37-1	1266	1286	snR40	1	20
77693-1_4	CACATCATGCATCACCATCT***TTTT***GTGGGTGGTGGTGCATG	-21.2	snR55	76	94	RDN37-1	1257	1277
-18530-1_2	GATCATGCATCACCATCTGATTAAGGATTGACAGATTGAG	-6.3	snR55	78	98	RDN37-2	1225	1243
125983-1_2	GA***TTTT***GTGGGTGGTGGTGCATGATCCGGCGATGATTCCTT	-6.8	RDN37-1	1255	1277	snR55 (D')	38	55
251846-1_1	GGA***TTT***GTGGGTGGTGGTGCAAATCATGCATCACCATCTGA	-19.9	RDN37-1	1258	1275	snR55 (D)	78	96
62794-1_6	G***TTTT***GTGGGTGGTGGTGCATAGCATCATGCATCACCATCT	-23.2	RDN37-1	1257	1279	snR55	77	94
310610-1_1	T***TTTT***GTGGGTGGTGGTGCATTGGAATATGTGCCATGGATT	-10.3	RDN37-1	1257	1276	snR55 (C')	55	75
301495-1_1	TGGGTGGTGGTGCATGGCCGTTCATGGATTACATCATGCAT	-8.9	RDN37-1	1262	1284	snR55	68	86
92915-1_3	CCGCGGTAATTCCAGCTCCAATAAAAACAAAAGCTGGAATT	-13	RDN37-1	572	594	snR77	20	38
99442-1_3	TGCGGTAATTCCAGCTCCAATAAAAGCTGGAATTACTGGCT	-20.6	RDN37-1	574	594	snR77	26	45
183610-1_1	CAGTAATTCCAGCTCCAATAAAAGCTGGAATTACTGGCTGA	-17.2	RDN37-1	577	594	snR77 (D')	26	47
32255-1_15	CGGTAATTCCAGCTCCAATAAAAGCTGGAATTACTGGCTGA	-20.4	RDN37-1	575	594	snR77 (D')	26	47
202069-1_1	CGGTAATTCCAGCTCCAATAAAAGCTGGAATTACTGGCTG	-20.4	RDN37-1	575	594	snR77	26	46
202068-1_1	CGGTAATTCCAGCTCCAATAAAAGCTGGAATTACTGGC	-20.4	RDN37-1	575	594	snR77	26	44
125360-1_2	GATGATCAGATACCGTCGTAGTTTTAATGATGATAGCATGA	-7.1	RDN37-1	994	1020	snR79 (C)	1	18
	**Sequence of hybrids between 25S rRNA and snoRNA**							
-10468-1_11	CTATTACAGTCGATGAGGATGATCGTCGAATTTGGGTATA	-9.8	snR4	3	22	RDN37-2 (snR40/snR60)	3438	3457

226958-1_1	GACGAATAAGGCGTCCTTGTACATTC***TT***AAGAATGACAAG	-3.5	RDN37-1	5731	5749	snR45	125	145
226959-1_1	GACGAATAAGGCGTCCTTGTACATTC***TT***AAGAATGACAAGG	-5.8	RDN37-1	5731	5749	snR45	125	146
114554-1_2	ACTGTTGGGACCGAAAGATGATTTAGTGATGATACTGCCG	-2	RDN37-1	3344	3363	snR39B (C)	1	20
92889-1_3	CCGAGCTTGACTCTAGTTTGACTCAAATGATGAAATACCAA	-5.6	RDN37-1	4963	4982	snR66 (C)	2	20
285819-1_1	TATAGGGGCGAAAGACTACTATCGAAATGAAGATAAAAATT	-4	RDN37-1	3454	3471	snR60 (C')	46	70
117007-1_2	CAAGTGGGAGCTTCGGCGCCACTATCGAAATGAAGATAAAA	-5.7	RDN37-1	5012	5031	snR60 (C')	47	67
	**Sequence of other hybrids with snoRNA**							
97363-1_3	TAAAGGGTTGCGCTTCTTTCCTGCGCAACCCA***TT***GATCTTG	-18.4	TEF1/2	1286	1304	snR45	100	123
**legend**	target guide box C, D, C', or D' Kre33-***Xlink*** site snR4-target							

‡ Reads are from the main data set (data-set II) and from a library with ~5-fold less coverage (*-dataset I*)

* predicted folding energy (kcal/mol) of the hybrid
